# *APOE* modulates microglial immunometabolism in response to age, amyloid pathology, and inflammatory challenge

**DOI:** 10.1016/j.celrep.2023.112196

**Published:** 2023-03-03

**Authors:** Sangderk Lee, Nicholas A. Devanney, Lesley R. Golden, Cathryn T. Smith, James L. Schwartz, Adeline E. Walsh, Harrison A. Clarke, Danielle S. Goulding, Elizabeth J. Allenger, Gabriella Morillo-Segovia, Cassi M. Friday, Amy A. Gorman, Tara R. Hawkinson, Steven M. MacLean, Holden C. Williams, Ramon C. Sun, Josh M. Morganti, Lance A. Johnson

**Affiliations:** 1 Department of Physiology, University of Kentucky, Lexington, KY 40536, USA; 2 Sanders Brown Center on Aging, University of Kentucky, Lexington, KY 40536, USA; 3 Department of Neuroscience, University of Kentucky, Lexington, KY 40536, USA; 4 Markey Cancer Center, University of Kentucky, Lexington, KY 40536, USA; 5 Department of Biochemistry & Molecular Biology, College of Medicine, University of Florida, Gainesville, FL, USA; 6 Center for Advanced Spatial Biomolecule Research, University of Florida, Gainesville, FL, USA; 7 These authors contributed equally; 8 Senior author; 9 Lead contact

## Abstract

The E4 allele of Apolipoprotein E (*APOE*) is associated with both metabolic dysfunction and a heightened proinflammatory response: two findings that may be intrinsically linked through the concept of immunometabolism. Here, we combined bulk, single-cell, and spatial transcriptomics with cell-specific and spatially resolved metabolic analyses in mice expressing human *APOE* to systematically address the role of *APOE* across age, neuroinflammation, and AD pathology. RNA sequencing (RNA-seq) highlighted immunometabolic changes across the *APOE4* glial transcriptome, specifically in subsets of metabolically distinct microglia enriched in the E4 brain during aging or following an inflammatory challenge. E4 microglia display increased *Hif1α* expression and a disrupted tricarboxylic acid (TCA) cycle and are inherently pro-glycolytic, while spatial transcriptomics and mass spectrometry imaging highlight an E4-specific response to amyloid that is characterized by widespread alterations in lipid metabolism. Taken together, our findings emphasize a central role for *APOE* in regulating microglial immunometabolism and provide valuable, interactive resources for discovery and validation research.

## In brief

Lee et al. integrate single-cell and spatially resolved -omics technologies to systematically characterize *APOE4*’s role in the brain’s response to aging, peripheral inflammatory challenge, and amyloid pathology. E4 microglia display a unique metabolic response to each of these paradigms, with increased aerobic glycolysis and altered expression of lipid metabolism pathways.

## Graphical Abstract

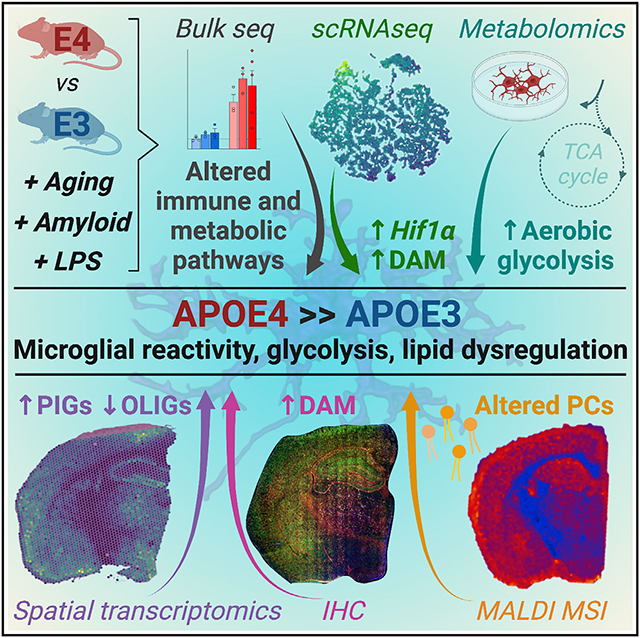

## INTRODUCTION

Metabolic dysfunction and chronic neuroinflammation are two features common to several neurodegenerative diseases, including Alzheimer’s disease (AD). Top hits from genome-wide association studies indicate that the microglial immune response is central to AD risk.^1-5^ Likewise, altered patterns of glucose and lipid metabolism are early biomarkers of incipient AD,^6^ with proteomic and metabolomic studies strongly linking changes in glial glucose metabolism to cognitive impairment and AD pathology.^7-10^ Tying metabolic dysfunction and neuroinflammation together is a well-established process whereby innate immune responses invoke metabolic reprogramming in microglia and vice versa.^11,12^ However, it remains unclear how this phenomenon of immunometabolism may relate to AD etiology and genetic risk factors.

Intriguingly, the strongest genetic risk factor for late-onset AD, the ε4 allele of Apolipoprotein E (*APOE*), has been separately linked to both heightened neuroinflammation and alterations in glial metabolism.^13^ In humans, there are three common alleles of *APOE*: ε2, ε3, and ε4. The ε4 allele is carried by nearly 20% of the population and confers up to a 15x increase in risk for AD compared with E3 homozygotes.^14^

Amyloid plaques trigger transcriptional changes in nearby microglia, inducing a shift toward a pathological signature.^15,16^ Similar neurodegenerative profiles have been described across several independent studies, being termed activated response microglia (ARMs),^15^ neurodegenerative microglia (MGnDs),^17^ or disease-associated microglia (DAMs).^18^ These signatures were initially described in mouse models with partial validation in human tissue. Interestingly, many of the genes within the aforementioned profiles belong to metabolic pathways, including core genes such as *Ch25h, Fabp5, Hexb, Lpl,* and *Apoe* itself.^19-24^ Although several studies attempted to translate these findings to postmortem human brain tissue using single-nucleus RNA sequencing (snRNA-seq), they found little overlap between human AD microglial gene signatures and those identified in mouse models.^25-27^ A glaring exception to this lack of overlap was *Apoe/APOE,* whose expression is amplified in neurodegenerative conditions across all studies and species, indicating that it is a universal, core transcriptomic “switch” within AD-associated microglia. However, it remains unclear whether isoform-specific differences in this process underlie the harmful effect of E4 in AD.

A handful of previous studies have examined the role of human *APOE* on the mouse brain transcriptome and metabolome,^28-32^ and a recent study inferred strong, glial-driven *APOE* genotype effects from whole-tissue bulk RNA-seq of postmortem human brains.^33^ Together these important studies highlight both amyloid-dependent and -independent roles for *APOE,* age, and brain region in metabolic and immune changes. However, their reliance on pre-selected brain regions and bulk homogenates limits insight into specific glial-cell-type contributions and lacks subregional anatomic resolution.

Here, we employed a single-source experimental design that combines bulk, single-cell, and spatial transcriptomics (ST) with cell-specific and spatially resolved metabolic analyses in order to systematically address the role of *APOE* across age, neuroinflammation, and AD pathology. Both bulk tissue and single-cell RNA-seq (scRNA-seq) highlighted immunometabolic changes across the *APOE4* glial transcriptome. Although aged E4 mice lack any observable AD pathology, we note that the gene signature expressed by their microglia (1) includes a robust increase in *APOE*; (2) is overrepresented by genes involved in glucose metabolism, lipid processing, and innate immunity; and (3) substantially overlaps with gene signatures previously described in both AD mouse models and human AD microglia. Further, exposing mice to a systemic inflammatory challenge resulted in a metabolically distinct response within E4 microglia. Using metabolomics and functional metabolic assays, we show that E4 microglia are inherently pro-glycolytic and *HIF1α*-high, displaying a metabolic profile associated with classically activated (M[lipopolysaccharide (LPS), interferon gamma (IFNg), tumor necrosis factor alpha (TNF-α)]) myeloid cells. We then crossed E3 and E4 mice to amyloid-overexpressing 5XFAD mice and utilized ST to determine anatomically salient changes in gene expression. ST highlighted the cortex and hippocampus as particularly sensitive to *APOE4* and revealed a unique response to amyloid in the E4 brain characterized by microglial activation and widespread alterations in lipid metabolism. Matrix-assisted laser desorption ionization (MALDI) mass spectrometry imaging (MSI) confirmed age-, *APOE-,* and region-specific alterations in lipid metabolism, particularly in multiple phospholipid species. Finally, we provide researchers with an interactive web-based resource (http://www.ljohnsonlab.com/database.html) in which to explore the effects of *APOE* across aging, neuroinflammation, and AD pathology via bulk, single-cell, and spatial transcriptomic datasets. Together, our findings link two phenomena consistently tied to AD (metabolic dysfunction and neuroinflammation) to the strongest genetic predictor of late-onset AD (E4), emphasizing a role for *APOE* in regulating glial immunometabolism.

## RESULTS

### *APOE4* drives immunometabolic changes across the glial transcriptome

**Figure 1.**
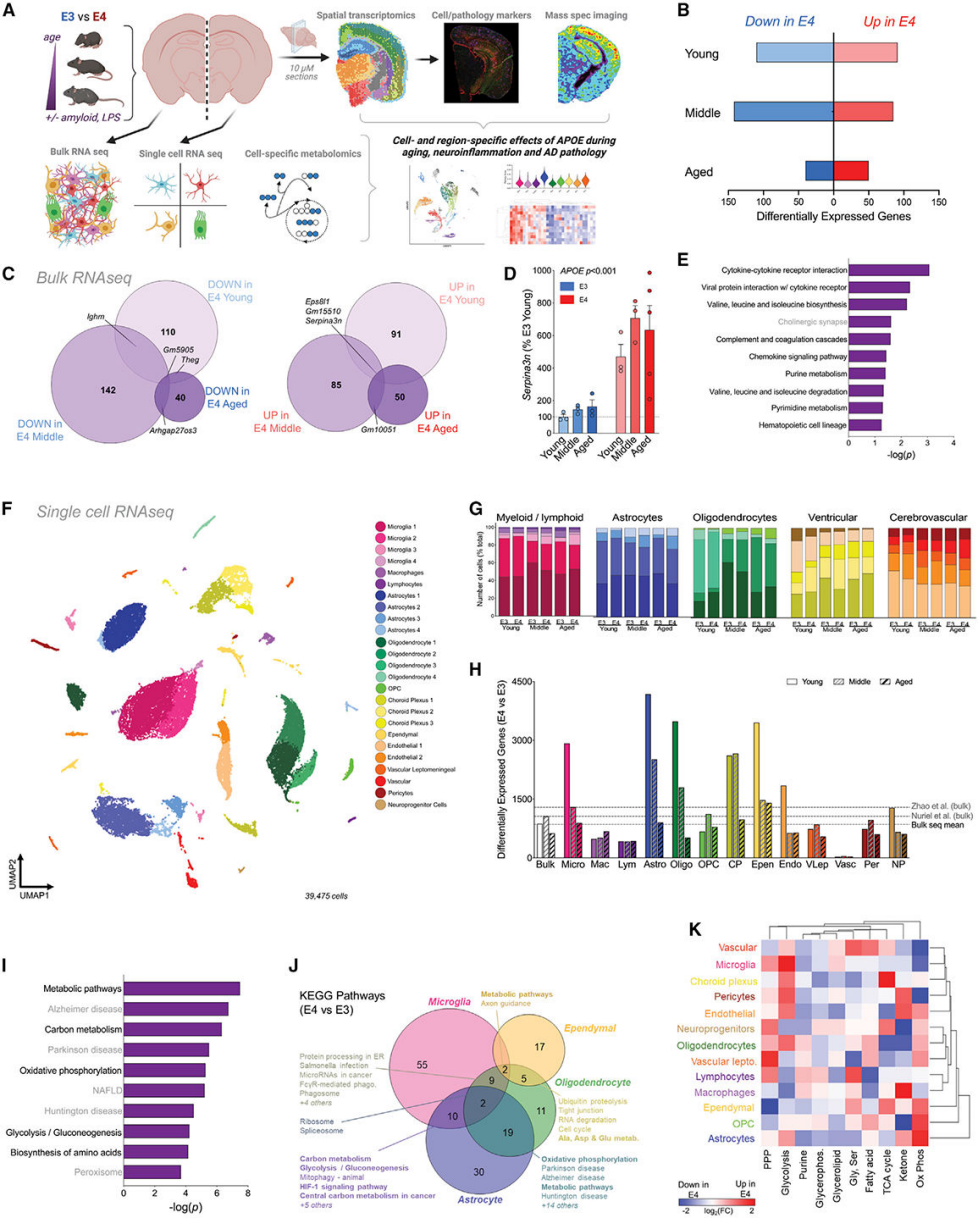
APOE4 drives immunometabolic changes across the glial transcriptome (A) Experimental design. Brains from APOE3 and APOE4 mice were analyzed across the lifespan (3, 12, and 24 months of age) and in the presence of an inflammatory challenge (LPS) or AD pathology (amyloid overexpression). (B and C) Number (B) and overlap (C) of differentially expressed genes (DEGs) (p < 0.01) between E3 and E4 brains at each age (bulk RNA-seq). Each circle is a comparison in young (light purple), middle-aged (purple), or aged (dark purple) mice; relative size corresponds to total DEGs. (D) Gene expression of *Serpina3n* in whole brain. *APOE* p < 0.001, two-way ANOVA. Error bars denote SEM. (E) The top 10 KEGG pathways most significantly altered by *APOE4* in whole-brain tissue. Terms in bold fall under KEGG umbrella pathways of “metabolism” or “immune system.” (F) UMAP showing 24 clusters classified based on canonical gene expression markers. (G) Number of cells per cluster. Bars are colored by individual cluster color from the UMAP in (F). (H) DEGs between E3 and E4 brains within each cell type at each age (scRNA-seq). Young, open bars; middle aged, gray dashed bars; aged, black dashed bars. Dashed lines indicate the number of DEGs in this, as well as two previous, bulk-seq analyses.^28,29^ (I) The top 10 KEGG pathways most significantly altered by *APOE4* across all cells (scRNA-seq). (J) Venn diagram showing overlap of KEGG pathways differentially expressed between E4 and E3 in the four cell types most affected by *APOE4*. Numbers represent number of significantly altered pathways in each cell type. The top five overlapping KEGG pathways are listed for each intersection. (K) Heatmap of the top 10 KEGG metabolic pathways altered by *APOE* in each cell type. Pathways in red show increased expression in E4 cells; blue indicates decreased expression. (B–K) Bulk-seq, n = 3–5 per group; scRNA-seq, 3 biological replicates were pooled together for n = 1 per experimental group. Glycerophos., glycerophospholipid metabolism; Gly,Ser, glycine and serine metabolism; OxPhos, oxidative phosphorylation; PPP, pentose phosphate pathway.

In order to systematically examine the effect of *APOE* genotype across aging, neuroinflammation, and AD pathology, we designed a single-source multi-omics approach that combined bulk- and scRNA-seq with cell-specific metabolomics and serial-section ST, histopathology, and MALDI MSI (Figure 1A). To examine the effects of *APOE* across the lifespan, we began with bulk sequencing of whole-brain tissue homogenates from young (3 months), middle-aged (12 months), and aged (24 months) mice expressing human E3 or E4 (n = 3–5). We identified a few hundred differentially expressed genes (DEGs) between E4 and E3 brains (Figures 1B and 1C), including previously reported genes such as *Serpina3n* (Figure 1D).^28,30^ To better understand these gene expression changes at a systems level, we performed a pathway analysis of E4 versus E3 DEGs. Nine out of the top 10 Kyoto Encyclopedia of Genes and Genomes (KEGG) terms fell under the umbrella pathways of “metabolism” or “immune system” (Figure 1E).

To identify cell-specific contributions to these whole-tissue gene expression changes, we performed scRNA-seq on the same brains analyzed for bulk sequencing (three pooled biological replicates for *n* = 1 per experimental group). Dimensionality reduction using uniform manifold approximation and projection (UMAP) identified 24 clusters that were assigned to 1 of 13 unique cell types using established gene markers (Figure 1F; Figure S1A). Although age-related enrichment of some clusters was observed, cell numbers were similarly distributed across *APOE* genotypes (Figure 1G; Figures S1B and S1C). Analysis of DEGs highlighted astrocytes, oligodendrocytes (OLIGs), ventricular cells, and microglia as the cell types most affected by *APOE* (Figure 1H). Similar to the bulk tissue, the number of DEGs decreased with age across several cell types (i.e., the effects of E4 were more pronounced in younger brains). A pathway analysis of DEGs across all cells together once more highlighted metabolism, particularly the central carbon metabolic pathways of oxidative phosphorylation (OxPhos) and glycolysis (Figure 1I). Further, KEGG pathways such as “metabolic pathways,” “glycolysis,” “OxPhos,” and “hypoxia inducible factor 1 (HIF-1) signaling” were differentially expressed across cell types (Figure 1J; Table S1).

Calculation of metabolic pathway activity for each individual cell using AUCell^34^ revealed the effect of E4 to be highly variable and cell specific. For example, astrocytes showed more pronounced E4-associated increases in branched-chain amino acid metabolism and OxPhos, while microglia displayed robust E4 increases in glycerolipid metabolism and glycolysis (Figure 1K). Together, these results suggest that the major transcriptomic changes driven by *APOE4* involve glial metabolism and the immune response.

### *APOE* expression is selectively upregulated in aged E4 microglia

We next asked whether expression of *APOE* itself varied across the lifespan in E3 and E4 glia. Although *APOE* expression did not vary by age or genotype in whole-brain tissue by bulk sequencing (bulk-seq) (Figure S2A), several changes were noted at the single-cell level. First, although *APOE* was predominantly expressed by astrocytes, many other cell types showed measurable levels of both *APOE* (Figures S2B-S2D) and its respective binding partners (Figures S2E, S2F, S3A, and S3B). Strikingly, whereas most cell types had subtle, if any, changes in *APOE* expression across the lifespan, aged E4 microglia showed a unique and dramatic upregulation *APOE* relative to E3 microglia (Figures S2G and S2H). Re-clustering of astrocytes and microglia across all ages showed that this upregulation was limited to a distinct sub-population of microglia (Mi_6) (Figures S2I-S2K and S3C). Finally, the *APOE* signaling network was differentially altered in both outgoing (*APOE*) and incoming (*Ldlr, Lrp1*, etc.) signal strength across these microglia and astrocyte sub-clusters in the aged E4 compared with aged E3 brain (Figure S3D). In summary, these findings highlight an age-related increase in *APOE* expression, in the absence of AD pathology, that is unique to E4 microglia.

### *Hif1α*-high, “DAM-like” microglia are increased in the aged E4 brain

**Figure 2.**
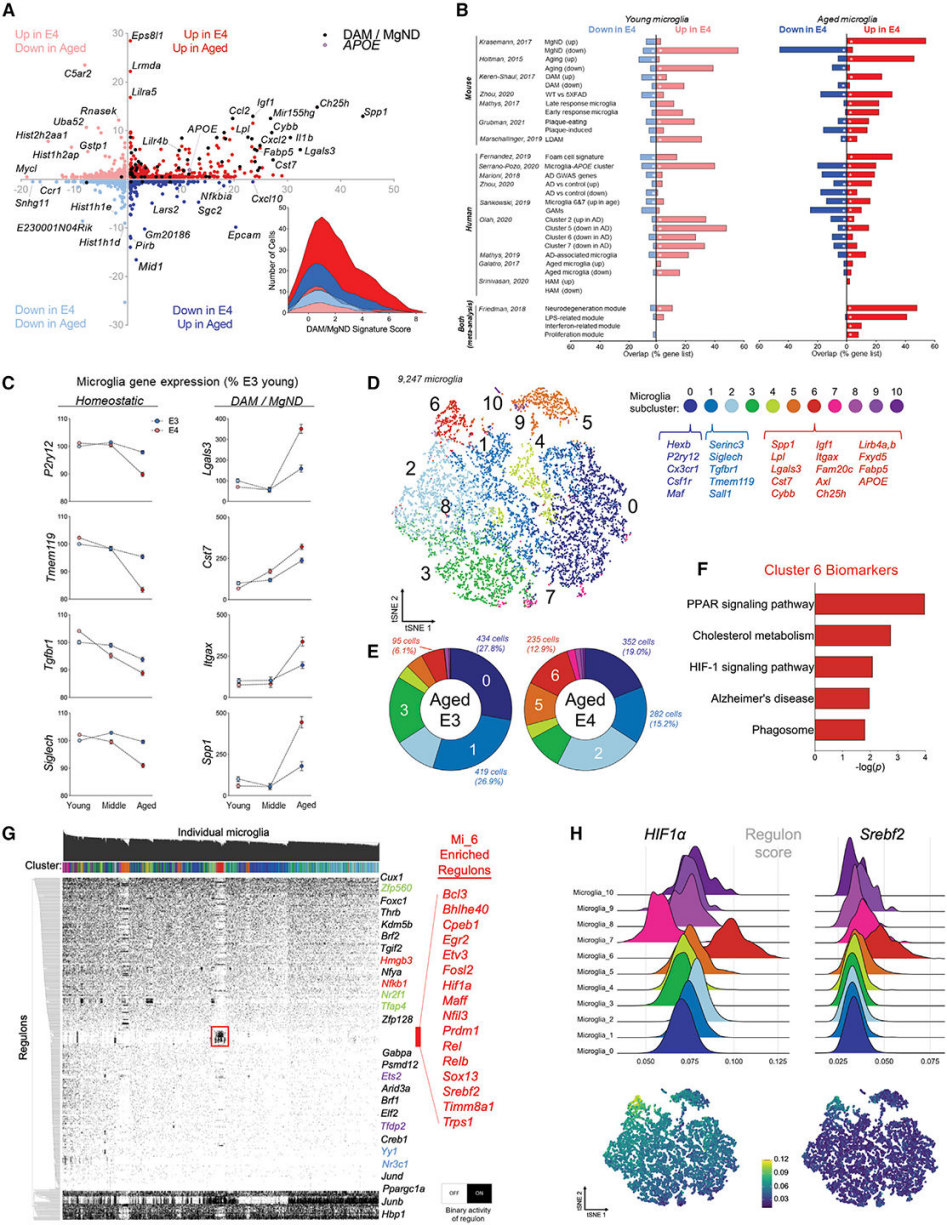
Age and *APOE4* are associated with an increase in “DAM-like” microglia (A) Gene score plot showing DEGs between E4 versus E3 microglia (y axis) and aged versus young microglia (x axis). Genes labeled in black are common to both DAM/MgND phenotypes. Inset: ridge plot showing DAM/MgND score for each individual microglia as calculated by AUCell. (B and C) E4-specific changes in the microglia transcriptome substantially overlap with AD-relevant gene lists from mouse and human studies. (B) Overlap of published gene lists with DEGs (E4 versus E3) in young (left) and aged (right) microglia (*p < 0.05, hypergeometric distribution test). (C) Expression of select “homeostatic” and DAM/MgND genes in young, middle-aged, and aged microglia. n = 1,422–1,951 cells/group. Error bars denote SEM. (D–F) Aged E4 microglia are enriched for a sub-cluster of cells with a DAM-like expression profile (cluster 6; Mi_6). (D) tSNE (t-distributed stochastic neighbor embedding) of microglia sub-clusters. Top biomarkers for the “homeostatic” clusters (0 and 1) and the “DAM-like” cluster 6 are displayed beneath the cluster labels. (E) Donut charts showing the distribution of aged E3 (left) and aged E4 (right) microglia within each sub-cluster. Clusters labeled in white are enriched in the respective group. (F) Top five Gene Ontology (GO) terms associated with the biomarkers that define Mi_6. (G) SCENIC was used to reconstruct active regulons in each individual microglia and meaningfully cluster cells based on shared activity patterns (binarized). Mi_6 is defined by selective high activity of 16 TFs (red box, “Mi_6 Enriched Regulons”) and the relative absence of activity of other TFs. (H) Ridge plots (top) or tSNE (bottom) showing regulon activity scores for *HIF1α* (left) and *Srebf2* (right).

To distinguish microglial genes that significantly change with age and/or E4, we calculated gene scores for each individual microglia. Strikingly, genes that are upregulated in both advanced age and E4 were heavily enriched for DAM/MGnD genes (Figure 2A). Further, E4-specific changes in the microglia transcriptome substantially overlapped with multiple AD-relevant gene lists from both mouse and human studies (Figure 2B; Table S1). Interestingly, we observed a “flip” in expression patterns for many AD-associated genes whereby young E4 microglia had higher expression, but aged E4 microglia had lower expression compared with E3, or vice versa (Figure 2C). Due to its unique upregulation of *APOE,* we next focused on microglia cluster 6 (Mi_6). Remarkably, the biomarkers that defined Mi_6 were almost exclusively genes associated with the DAM/MGnD signature, including metabolic genes *Lpl, Ch25h, Fabp5,* and *APOE* itself (Figure 2D; Table S1). Mi_6 was enriched in aged E4 mice (12.9% of all microglia) relative to E3 (6.1%) (Figure 2E; Figures S4A and S4B), and a pathway analysis of the Mi_6 biomarkers highlighted “Alzheimer’s disease” and metabolic pathways including “cholesterol metabolism” and “HIF1 signaling” (Figure 2F).

In order to identify potential upstream regulators that define these various microglia clusters, we used SCENIC (Single-Cell Regulatory Network Inference and Clustering) to reconstruct active regulons (i.e., transcription factors [TFs] and their target genes) in individual microglia.^34^ SCENIC revealed a clear and distinctive clustering of Mi_6 defined by 16 regulons (Figures 2G and 2H). Intriguingly, several of these regulons have been previously implicated in AD (*Bhlhe40*),^17^ regulate metabolic pathways (*Timm8a1*,^35^ *Srebf2*^36^), or both (*Hif1α*^37^) (Figures 2G and 2H). *HIF1α* in particular was substantially upregulated in Mi_6 (Figure 2H) and was positively correlated with the cell’s DAM/MGnD score (Figure S4C). Given the central role of these regulons in metabolism, we next sought to characterize metabolic activity within each cluster. A heatmap of metabolic pathway scores revealed Mi_6 as the cluster with the highest expression of central carbon pathways, including glycolysis (Figures S4D and S4E). Together, these data show that even in the absence of overt AD pathology, age and E4 are sufficient to drive changes in microglia that (1) overlap with both mouse and human AD-relevant gene lists, (2) strongly resemble a DAM phenotype, and (3) prominently feature distinct shifts in the regulation of glucose and lipid metabolism.

### E4 microglia have a distinct metabolic response to an inflammatory challenge

**Figure 3.**
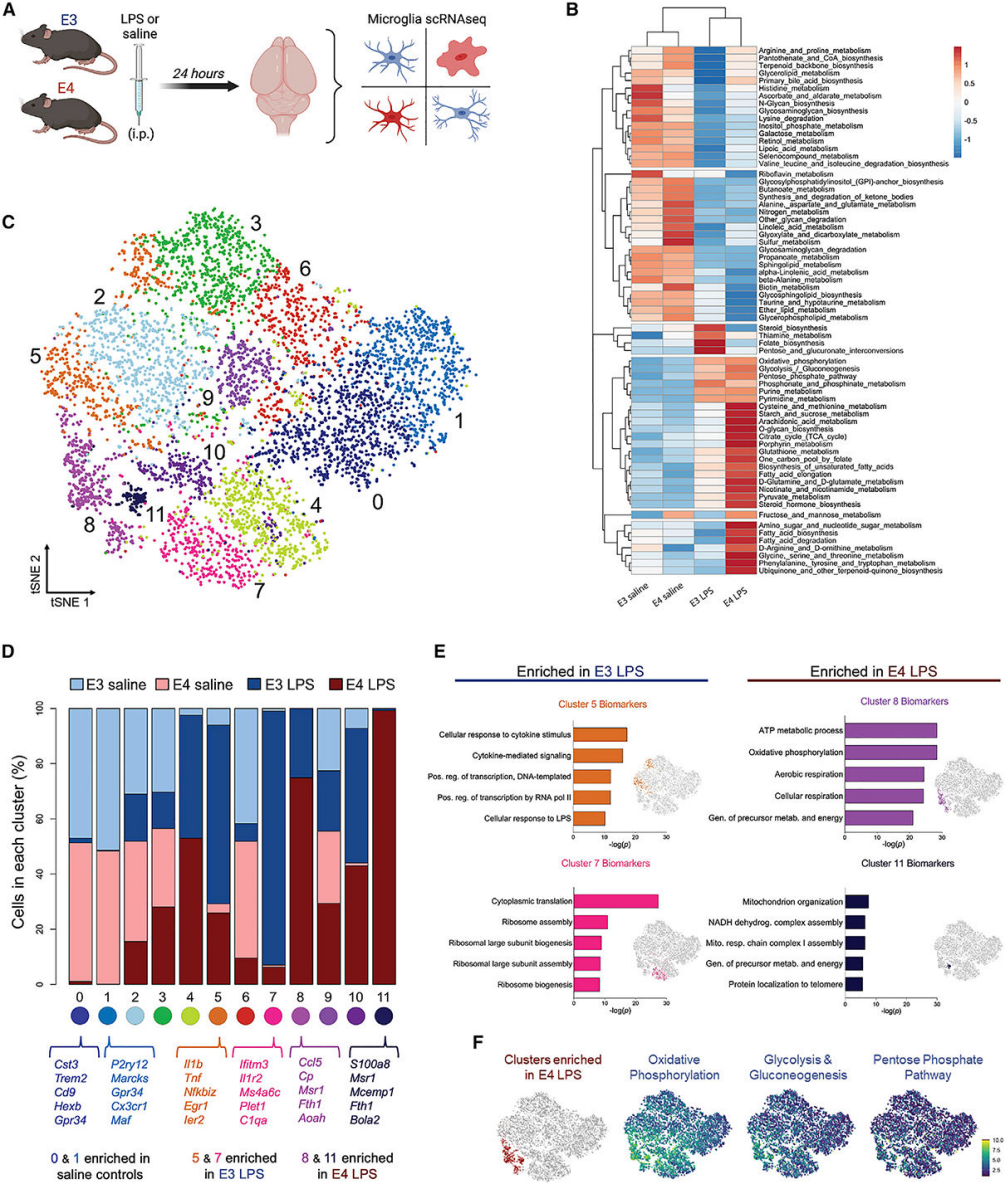
APOE4 microglia are metabolically distinct in response to an inflammatory challenge (A) Experimental design. E3 and E4 mice were injected with lipopolysaccharide (LPS; 5 mg/kg) or saline, and brains were dissected 24 h later for scRNA-seq. (B) Heatmap showing expression of KEGG metabolic pathways in microglia from LPS- or saline-treated mice. (C and D) E3 and E4 brains show enrichment of distinct microglia sub-clusters following LPS treatment. (C) tSNE plot of microglia from LPS- or saline-treated E3 and E4 mice. Colors highlight the 12 microglia sub-clusters. (D) Stacked bar plot showing distribution of experimental groups within each microglia sub-cluster. Top five biomarkers for the two “homeostatic” (0 and 1), E3-enriched (5 and 7), and E4-enriched (8 and 11) clusters are listed below. (E and F) E4 LPS microglia are associated with energy production and OxPhos pathways. (E) Top five GO terms associated with the two E3 LPS-enriched (left, 5 and 7) and two E4 LPS-enriched (right, 8 and 11) clusters. (F) tSNE plots showing higher expression of central carbon (i.e., energy production) pathways in sub-clusters enriched in the E4 LPS brain. Three biological replicates were pooled together for n = 1 per experimental group.

Given their unique metabolic transcription profile and DAM-like signature, we next asked whether E4-expressing microglia would differentially respond to an inflammatory challenge. Twenty-four hours following a peripheral injection of LPS or saline, we harvested brains from E3 and E4 mice (three pooled biological replicates for *n* = 1 per experimental group) and performed scRNA-seq on microglial populations (Figure 3A). Microglia from the E4 LPS brains showed a remarkably distinct metabolic profile, with increased activity across multiple pathways of amino acid, sugar, and fatty acid metabolism (Figure 3B). At the subpopulation level, treatment with LPS resulted in several distinct clusters of microglia, including two clusters enriched in E3 LPS brains (5 and 7) and two found almost exclusively in E4 LPS brains (8 and 11) (Figures 3C and 3D). Notably, the DEGs defining these E4 LPS-enriched clusters correspond to Gene Ontology terms related to mitochondrial function, aerobic respiration, and energy production (Figure 3E). These E4 LPS-enriched clusters also showed high expression of genes belonging to OxPhos and glycolysis pathways (Figure 3F). In total, these data suggest that compared with E3, expression of E4 leads to a robust and distinct metabolic response by microglia to an inflammatory challenge.

### E4 microglia have increased aerobic glycolysis and higher *HIF1α* expression

**Figure 4.**
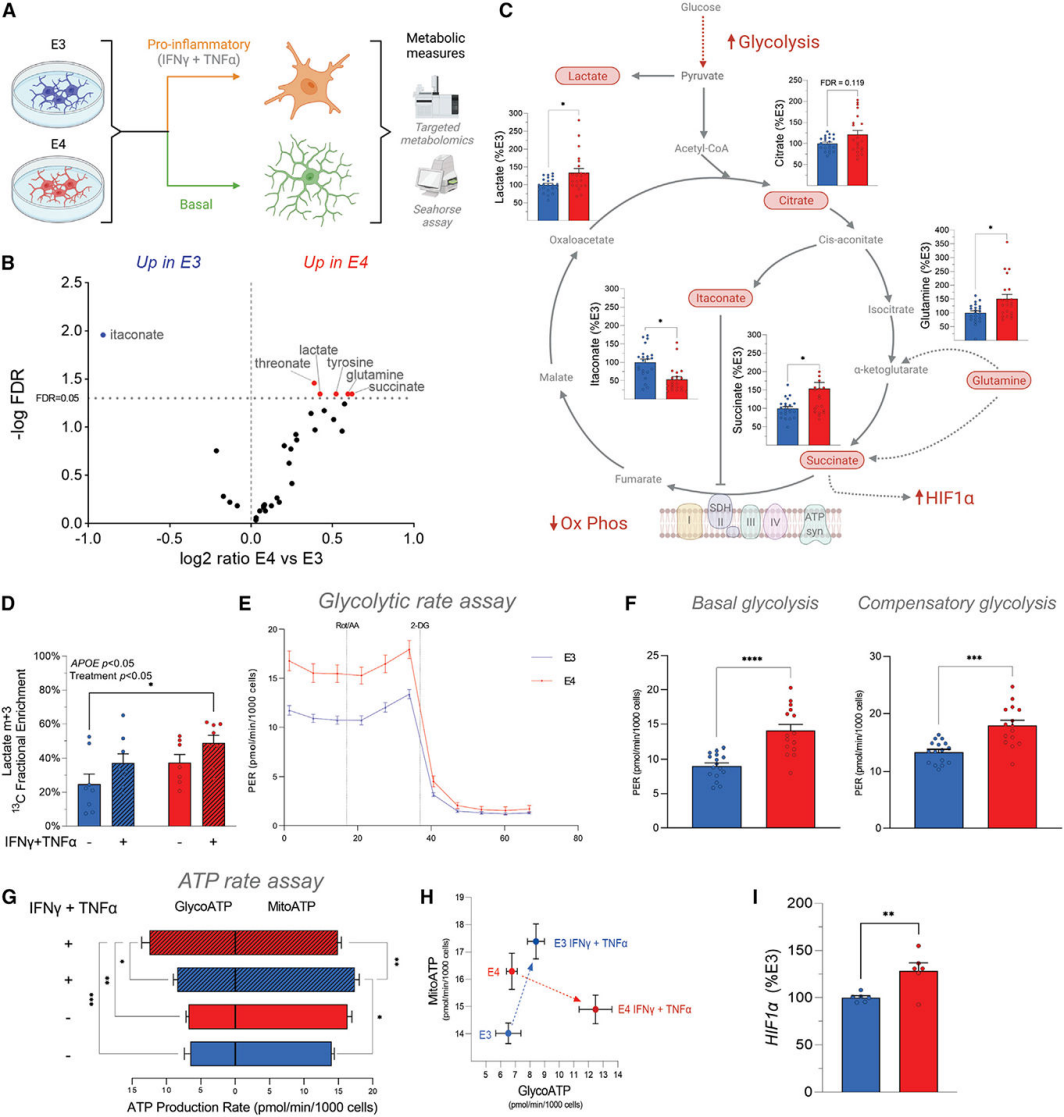
E4 microglia have increased aerobic glycolysis and higher *Hif1α* expression (A) Experimental design. Primary microglia were isolated from E3 and E4 mice and stimulated *in vitro* with a pro-inflammatory (20 ng/mL IFNγ +50 ng/mL TNF-α) cytokine cocktail prior to Seahorse analysis or targeted metabolomics (both steady-state and stable-isotope-resolved metabolomics). (B and C) Targeted metabolomics on E3 and E4 microglia (*n* = 21–22 per group). (B) Volcano plot showing changes in steady-state metabolites. (C) Schematic of TCA cycle and glycolysis. Pathways and metabolites associated with pro-inflammatory immunometabolism are highlighted in red, with corresponding bar graphs for E3 and E4 steady-state metabolites overlaid on each. (D) Stable-isotope tracing reveals increased fractional enrichment of fully labeled (m+3) lactate in pro-inflammatory-treated E4 microglia (*n* = 7–8 per group) after 2 h. (E) Proton efflux rate (PER) (pmol/min/1,000 cells), a measure of glycolysis, measured over time in E3 and E4 microglia during the glycolytic rate assay (Agilent). (F) E4 microglia showed higher basal glycolysis (left) and compensatory glycolysis (right) compared with E3 controls (n = 15–16 per group). (G) ATP production rate (pmol/min/1,000 cells) measured during the ATP rate assay (Agilent) in E3 and E4 microglia, with glycolytic ATP production (GlycoATP) to the left of the y axis and mitochondrial ATP production (MitoATP) to the right (n = 5–9 per group). (H) xy plot with MitoATP displayed on y axis and GlycoATP displayed on x axis. E4 microglia respond to stimulus by dramatically increasing GlycoATP and decreasing MitoATP (red dashed arrow), whereas E3 microglia respond with only a slight increase to GlycoATP and instead show a dramatic increase in MitoATP (blue dashed arrow). (I) Quantitative RT-PCR analysis shows increased *Hif1α* gene expression in E4 primary microglia (n = 6 per group). Error bars denote SEM. *p < 0.05, **p < 0.01, ***p < 0.001, ****p < 0.0001. Two-way ANOVA (D and G), two-tailed t test (F and I), or two-tailed t test adjusted for multiple comparisons (indicated as FDR [false discovery rate]) (B and C).

We next sought to determine whether these differences in gene expression would be functionally reflected in altered metabolism between E4 and E3 primary microglia (Figure 4A). Using a targeted metabolomics approach, we identified five metabolites that were significantly upregulated in E4 microglia (lactate, succinate, glutamine, tyrosine, and threonate) and one significantly downregulated (itaconate) (n = 21–22) (Figures 4B and 4C; Figure S5A and S5B). Notably, lactate accumulates in cells undergoing increased aerobic glycolysis, such as pro-inflammatory activated macrophages.^11,12^ Citrate and succinate also accumulate in pro-inflammatory macrophages because of a break in the TCA cycle.^11,12^ Succinate was significantly increased in E4 microglia, whereas concentrations of itaconate, which activates downstream anti-inflammatory and antioxidant signaling pathways,^38-40^ were lower (Figures 4B and 4C).

To ascertain whether these differences in steady-state metabolite pool sizes were part of a more dynamic alteration in metabolic flux within E4 microglia, we next turned to stable-isotope-resolved metabolomics. E3 and E4 microglia were stimulated with a combination of IFNγ and TNF-α in the presence of [^13^C]glucose, and incorporation of ^13^C in downstream metabolites was measured. This revealed a significant increase in fully labeled (m+3) [^13^C] lactate as a result of both *APOE4* and pro-inflammatory treatment, indicating increased flux of glucose through aerobic glycolysis (*n* = 7–8) (Figure 4D).

To functionally assess the effect of *APOE* on microglial metabolism, we employed the Seahorse platform to measure glycolysis, mitochondrial respiration, and the relative contribution of each pathway to ATP production. Interestingly, we noted that E4 microglia showed higher rates of basal and compensatory glycolysis compared with E3 (Figures 4E and 4F) and had lower maximal respiration and spare respiratory capacity (n = 15–16) (Figures S5C-S5F). In addition, E4 microglia responded to a proinflammatory stimulus by dramatically increasing glycolytic ATP production at the expense of decreased mitochondrial production. In contrast, E3 microglia significantly increased mitochondrial ATP production following stimulation (Figures 4G and 4H). These data suggest that E4 microglia rely exclusively on a substantial upregulation of glycolysis to support the increased energy demand of the pro-inflammatory response, whereas E3 microglia demonstrate increased metabolic flexibility.

Finally, the increased succinate and clear functional shift toward aerobic glycolysis in E4 microglia is congruent with the increased activity of the *Hif1α* regulon in the E4 microglia SCENIC data. When stabilized by a pro-inflammatory stimulus (and/or succinate), the *HIF1α* TF complex translocates to the nucleus and activates many genes important for increasing glycolysis.^41^ In agreement with this pro-glycolytic E4 phenotype, quantitative RT-PCR revealed increased expression of *Hif1α* in E4 microglia compared with E3 (*n* = 6) (Figure 4I). Together these data highlight functional metabolic reprogramming whereby E4 microglia are inherently pro-glycolytic and anti-oxidative, a phenotype that mirrors classically activated macrophages.

### ST identifies unique cortical and hippocampal signatures of *APOE4,* age, and amyloid overexpression

**Figure 5.**
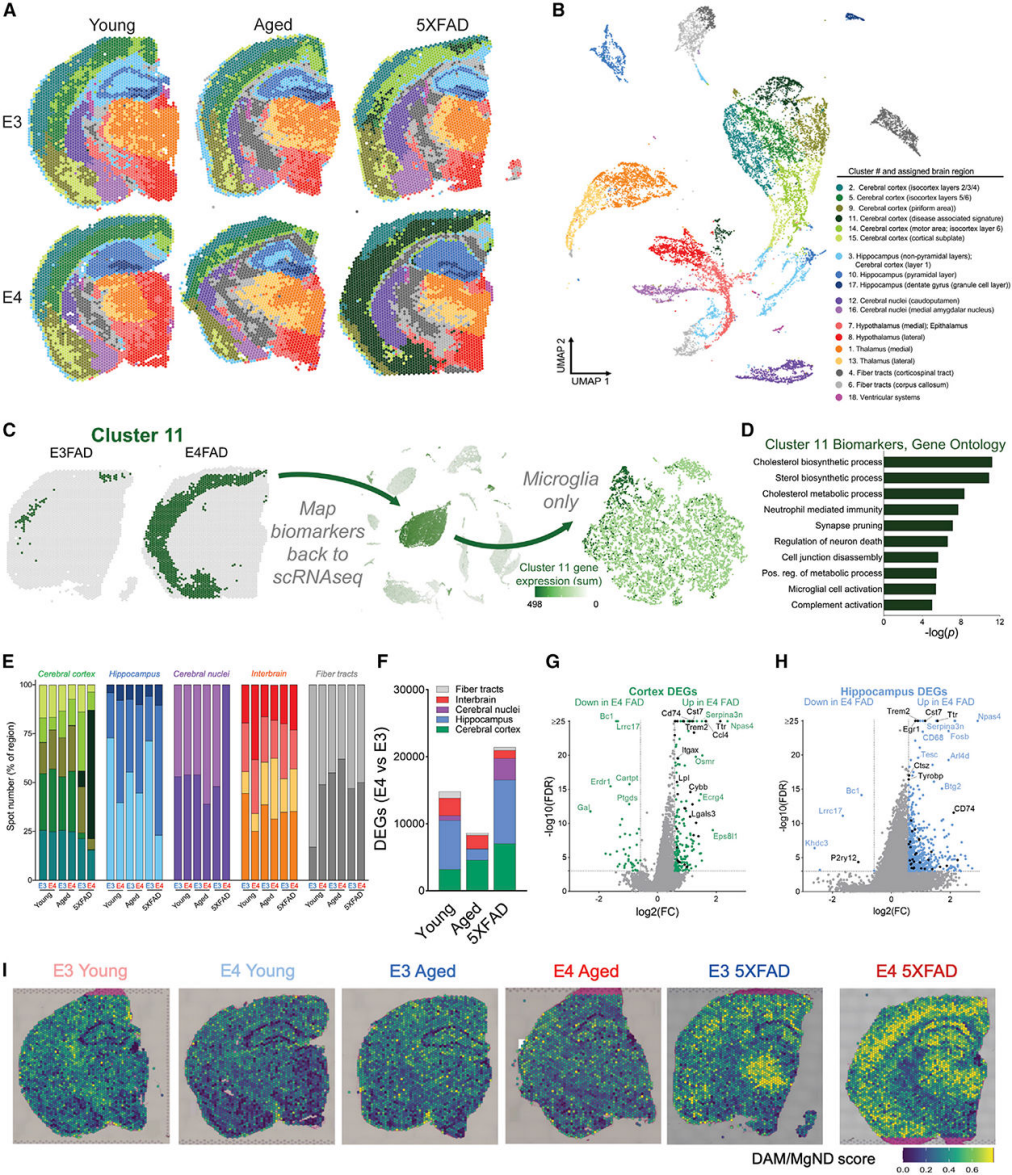
Spatial transcriptomics (ST) highlights unique cortical and hippocampal signatures of *APOE4*, age, and amyloid overexpression (A and B) ST identifies 17 unique clusters that are anatomically conserved, plus one unique cortical cluster primarily restricted to E4FAD mice (cluster 11, dark green). (A) Spatial transcriptomic plots of brain sections from young, aged, and amyloid-overexpressing E3 and E4 mice. (B) UMAP plot of all 16,979 spots analyzed across all six brains. Clusters were assigned labels based on anatomical concurrence to the Allen Brain Atlas. (C and D) Cluster 11 is enriched in the E4FAD brain and consists of genes related to lipid metabolism and microglial activation. (C) E3FAD and E4FAD brains showing spots belonging to cluster 11. Cluster 11 biomarker genes were re-plotted to scRNA-seq data, showing highest expression in microglia, specifically in Mi_6. (D) Top 10 Gene Ontology terms for cluster 11, highlighting pathways of lipid metabolism and immune activation. (E) Number of spots within each cluster for each experimental group. Clusters are organized by respective brain regions. (F–H) E4 drives gene expression changes primarily in the cortex and hippocampus. (F) DEGs between E4 and E3 brains within each brain region. (G and H) DEGs within the cortex (G) and hippocampus (H) of the 5XFAD mice. Genes labeled in black correspond to DAM/MgND genes. (I) ST plots showing DAM/MgND scores for each spot (calculated with AUCell). n = 1 brain per group.

We next leveraged Visium ST technology to assess gene expression across coronal brain sections from E3 or E4 mice at young and old ages compared with E3 or E4 mice crossed to the 5XFAD amyloid-overexpressing mouse model (Figure 5A; Figure S6A). A total of 16,979 spots were analyzed across six brains, and high dimensionality reduction identified 18 total clusters. This included 17 anatomically conserved clusters that expressed canonical region-specific markers and visually mapped to broad regions of the Allen Brain Atlas (Figures 5A and 5B; Figures S6B and S6C). Intriguingly, the final cluster (cluster 11) was found almost exclusively in the E4 5XFAD brain and was primarily localized within cortical regions (Figure 5C). This cluster, which we term a “disease-associated” signature, was defined by biomarkers enriched in pathways related to lipid metabolism, synapse pruning, neuronal death, and microglial activation (Figure 5D). When we mapped these cluster 11 biomarkers back to our scRNA-seq data, the signature was exclusively and highly expressed by microglia, with the Mi_6 cluster showing peak expression (Figure 5C).

We next assigned spots to one of five primary brain regions (cerebral cortex, hippocampus, cerebral nuclei, interbrain, or fiber tracts), noting that the majority of E4 versus E3 DEGs were found in the cerebral cortex and hippocampus (Figures 5E and 5F; Figures S7A-S7C). Both regions featured a robust upregulation of genes in the E4 5XFAD compared with E3 5XFAD brain, many of which were DAM/MGnD genes (Figures 5G-5I). In addition, gene markers of glial reactivity previously linked to *APOE4* were similarly upregulated in E4 aged and E4 5XFAD brains (Figure S7D). Taken together, these results (1) support an E4-associated increase in microglial activation, (2) highlight cortical and hippocampal regions as areas most affected by *APOE*, and (3) reveal a unique E4 response to amyloid pathology.

### *APOE4* exacerbates plaque-induced microglial activation and lipid metabolism

**Figure 6.**
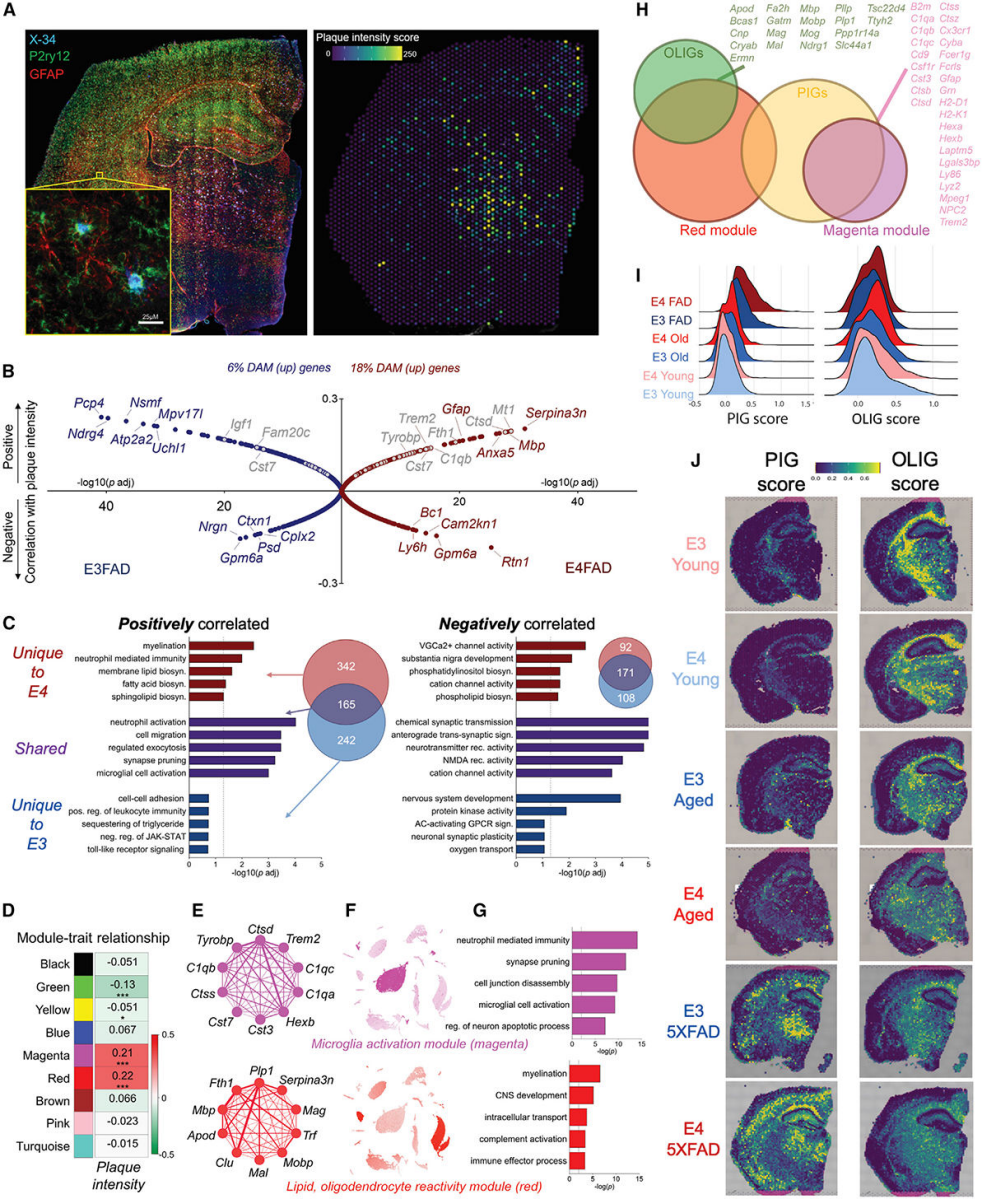
*APOE4* exacerbates plaque-induced microglial activation and alterations in lipid metabolism (A) E4FAD brain stained with P2ry12 (green; microglia), GFAP (red; astrocytes), and X-34 (blue) to demarcate amyloid plaques (left). X-34 intensity was quantified to generate a “plaque intensity score” for each individual spatial transcriptomic spot (right). (B) Gene correlation with plaque intensity in E3FAD (blue, left) and E4FAD (red, right) brains. y axis values represent correlation coefficients, with genes at the top of the graph positively correlated with plaque intensity, and genes at the bottom negatively correlated. Distance from center on the x axis represents significance of the correlation (−log10(p adjusted)). DAM/MgND genes are noted in gray. (C) Top five Gene Ontology terms for genes that were positively (left) or negatively (right) correlated with plaque intensity. Some GO terms were uniquely correlated with E4 (red), some uniquely correlated with E3 (blue), and some correlated with plaque intensity regardless of *APOE* genotype (purple). Venn diagrams show overlap between genes correlated with plaque intensity in E4FAD (red circles) or E3FAD (blue circles) brains. (D–G) Gene networks associated with plaque intensity. (D) The correlation between module eigengenes (MEs) and amyloid plaque intensity. Values in the heatmap are Pearson’s correlation coefficients, and asterisks represent significant correlations: *p < 0.05; ***p < 0.001. Modules with positive values (red) indicate positive correlation of MEs with plaque intensity, modules with negative values (green) represent a negative correlation. (E) Network plots of the top 10 genes with the highest intramodular connectivity (hub genes) in the magenta (top) and red (bottom) modules. (F) UMAP plots map expression of module gene lists (sum) back to the scRNA-seq dataset. (G) Top five Gene Ontology terms associated with the magenta or red modules. (H) Venn diagrams showing overlap of red and magenta modules with oligodendrocyte (OLIG) and plaque-induced gene (PIG) lists from Chen et al.^16^ Overlapping genes are listed. (I and J) The E4FAD brain has a high PIG score and the lowest OLIG score. (I) Ridge plots showing PIG (left) and OLIG (right) scores for each experimental group. (J) Spatial expression of PIG (left) and OLIG (right) gene lists.

To determine whether this unique E4 5XFAD transcriptional profile was spatially linked to AD pathology, we stained for amyloid plaques across the 10-μM section immediately adjacent to that subjected to ST, and we assigned each spot a numerical plaque intensity score (Figure 6A). A correlation analysis revealed a number of genes that either positively or negatively tracked with plaque intensity. Notably, in the E4 brain, this included strong positive correlations with markers of glial reactivity and a 3-fold increase in the number of significantly correlated DAM genes (Figure 6B). A pathway analysis of significantly correlated genes showed numerous terms shared by both E3 and E4 (purple), as well as some unique to E4 (red) or E3 (blue) (Figure 6C). Shared pathways included terms related to synaptic transmission (negatively correlated) or synapse pruning and microglial activation (positively correlated) (Figure 6C). Interestingly, pathways unique to E4 were predominantly related to lipid metabolism (Figure 6C).

A weighted gene co-expression network analysis (WGCNA) highlighted two networks (green, yellow) containing genes related to ion channels and synaptic transmission that were negatively associated with plaque intensity and two networks (magenta, red) that were positively associated (Figures 6D-6G; Figures S8A and S8B). The magenta network we termed a “microglia activation module,” because it mapped almost exclusively to microglia in our scRNA-seq database, was enriched for DAM genes and was associated with Gene Ontology terms related to synapse pruning, neuron apoptosis, and microglia activation (Figures 6E-6G, top). In contrast, the red “lipid, oligodendrocyte reactivity” module mapped predominantly to OLIGs, and it included markers of lipoprotein transport, myelin, and glial reactivity (Figures 6E-6G, bottom).

Both the red and magenta modules were more highly expressed across the E4 5XFAD brain relative to E3 (Figure S8A), and intriguingly they substantially overlapped with the OLIG and plaque-induced gene (PIG) networks identified from a previous ST study of AD mouse and human brains^16^ (Figure 6H). Interestingly, the PIG score was lowest in the young brains, increased slightly with age, and was highest in the E4 5XFAD brain, with the OLIG scoring following the opposite trend (Figures 6I and 6J). In summary, these data highlight a unique E4 response to increasing amyloid pathology characterized by increased microglial activation and alterations in lipoprotein and lipid metabolism.

### MALDI MSI confirms *APOE-,* age-, and amyloid-associated changes in lipid metabolism

**Figure 7.**
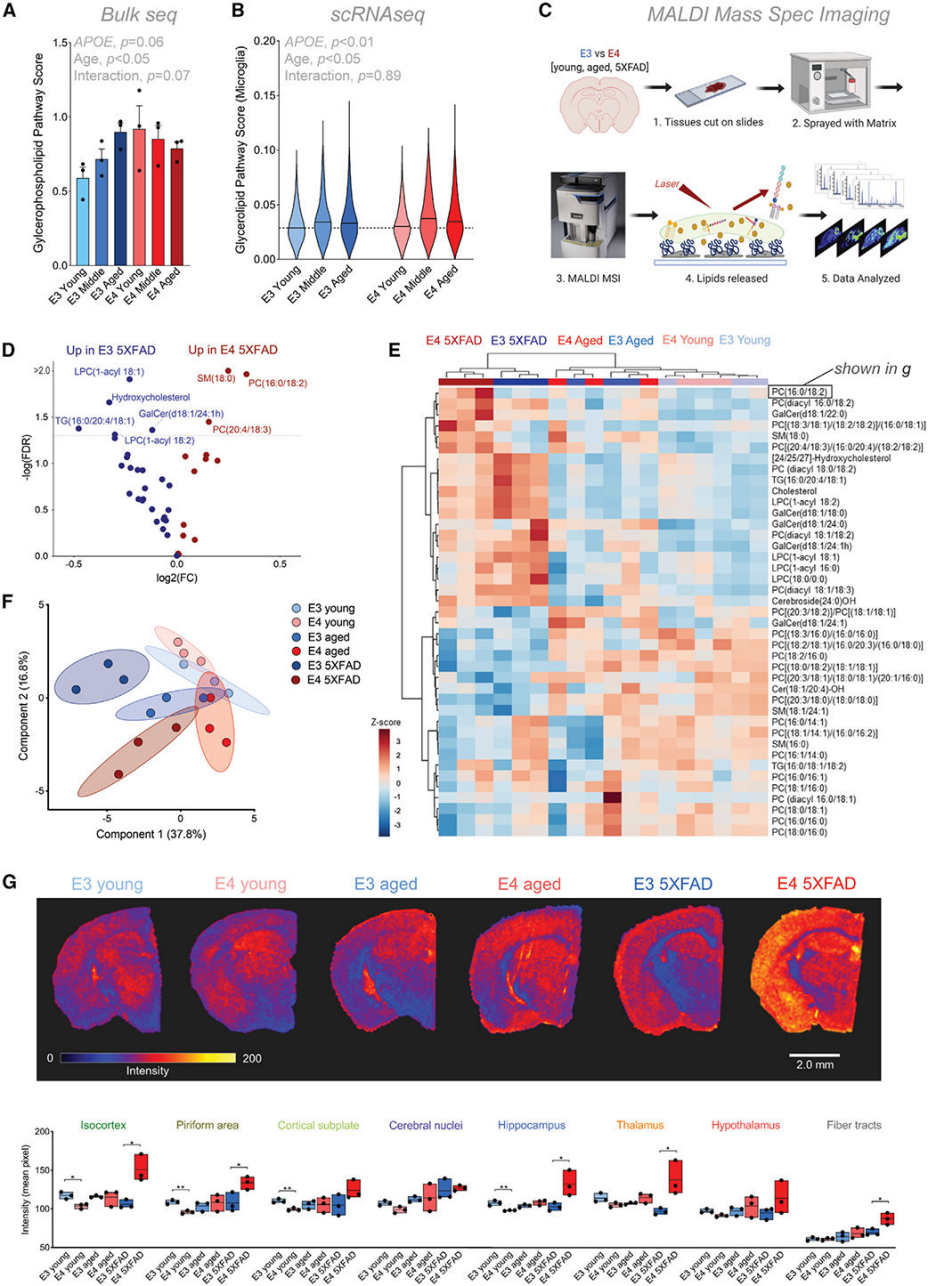
Matrix-assisted laser desorption ionization (MALDI) mass spectrometry imaging (MSI) reveals *APOE*- and region-specific changes in multiple lipid species (A and B) Expression of glycerophospholipid pathway genes increases with age in whole-brain tissue (A, ‘*bulk*’) and is highest in aged E4 microglia (B, ‘*scRNA-seq*’). (C) Experimental workflow for MALDI MSI. (D) Volcano plot of targeted lipid species highlights changes in select phosphatidylcholine, sphingomyelin, ceramide, and triacylglycerol. (E) Heatmap of quantified lipid species (average values across all regions) shows clear clustering by age and amyloid expression, with distinct separation of E3 5XFAD and E4 5XFAD brains. Brackets include multiple possible fatty acid chain lengths and/or double-bond positions. (F) Principal-component analysis (PCA) plot of MALDI MSI-detected lipids shows clear separation of E3 5XFAD and E4 5XFAD brains. (G) Regional intensity of an example lipid from (E) (phosphatidylcholine (16:0/18:2)). (Top) Scans show spatial distribution of lipid across coronal brain sections. (Bottom) Average pixel intensity across each brain region for PC(16:0/18:2). n = 3 per group. Error bars denote SEM. *p < 0.05, **p < 0.01, multiple comparisons ANOVA. Regional data for all scanned lipids can be found in Table S2.

In whole-brain tissue, we noted significant effects of *APOE,* age, and their interaction on the expression of multiple lipid metabolism pathways (Figure 7A; Figure S9A). At the single-cell level, changes in lipid metabolism were most pronounced in microglia, specifically in glycerophospholipids, with aged E4 microglia having the highest expression (Figure 7B).

In order to validate the gene signatures implicating lipid dysregulation, we turned to MALDI MSI to generate qualitative, spatially resolved measures of targeted lipid species (n = 3) (Figure 7C). Following fine-spatial scans of coronal sections, we assigned each MALDI MSI pixel to one of the anatomically assigned regions. The overall lipid profiles showed clear heterogeneity, with samples generally clustering well by anatomical region (Figure S9B). The primary exception was the “disease-associated” area found almost exclusively in the E4 5XFAD brain. This region did not clearly cluster with itself nor with any other specific anatomical region, suggesting widespread dysregulation of lipid metabolism (Figure S9B).

The concentrations of multiple lipid species were altered in the E4 5XFAD brain relative to other groups, including multiple phosphatidylcholines (PCs), which are a subtype of glycerophospholipid (Figures 7D and 7E). Clustering analyses showed distinct separation of the E3 5XFAD and E4 5XFAD brains relative to other groups and to each other (Figure 7F). Interestingly, many of the observed age-, amyloid-, and *APOE*-associated changes in lipid concentrations were regionally specific (Table S2). For example, the PC most increased in the E4 5XFAD brain relative to E3 5XFAD (PC (16:0/18:2)) showed dramatic changes in the isocortex, hippocampus, and thalamus, but no difference in the piriform area, cortical subplate, and hypothalamus (Figure 7G). Together, these results show that the transcriptional signatures implicating dysregulation of lipid metabolism in the E4 5XFAD brain are validated by alterations in multiple lipid species, in particular, PCs.

## DISCUSSION

It is increasingly appreciated that chronic neuroinflammation and metabolic dysfunction are early and prominent actors over the course of AD.^42-44^ Notably, these two features are innately linked through the concept of immunometabolism.^11,12^ Microglia are highly metabolically active cells^45^ that play a central role in maintaining CNS immune homeostasis, and the majority of genetic risk factors associated with late-onset AD are highly or specifically expressed in this cell type.^46^ Many of these, including the strongest genetic risk factor for late-onset AD, *APOE,* are thought to integrate metabolic inputs with downstream inflammatory signaling.^47-49^ Here, we set out to systematically study the impact of *APOE* genotype across age, inflammatory challenge, and in response to amyloid using an integrative multi-omics approach. Collectively, our findings implicate*APOE4* as a driver of a dysfunctional immunometabolic response across each condition.

### A single-cell view of *APOE* immunometabolism

Our “bulk” tissue sequencing highlighted brain-wide changes in multiple immune and metabolic pathways similar to previous studies.^28,30^ Although these data pointed toward E4-associated increases in metabolic, cytokine/chemokine, and complement pathways across the whole tissue landscape, the cell-specific changes potentially driving these bulk responses remained unknown. Therefore, we leveraged a scRNA-seq approach using the same tissue samples analyzed for “bulk” sequencing. In doing so, we identified a unique enrichment of a population of microglia, Mi_6, that predominates in the aged E4 brain. Differentially expressed biomarkers for this sub-cluster were enriched for genes involved in lipid metabolism and the innate immune response, as well as markers associated with DAM staging of microglia. Canonically, these populations emerge in response to neurodegenerative insults such as amyloid pathology, demyelination, or phagocytosis of apoptotic neurons.^17,18^ It is therefore striking that a similar population of microglia (Mi_6) appears in aged E4 brains even while they lack discernible pathology.

These cell-type- and subpopulation-specific differences appear to be important in understanding cross-species relevance. For example, Serrano-Pozo et al.^33^ recently identified a transcriptional signature associated with E4 carriage in patients with AD. Interestingly, when we compare this gene list with our scRNA-seq data, over 50% of the pro-inflammatory and phagocytic genes upregulated in the brains of E4^+^ individuals with AD were significantly downregulated in young E4 microglia. This “flip” in E4 microglia gene expression from lower in young to higher in aged was observed across many other gene lists from both mouse models of AD and human AD microglia studies. Decreased expression of one of these genes, *Lgals3* (galectin-3), was recently found to protect against retinal ganglion cell (RGC) loss, with E4 microglia failing to upregulate *Lgals3* and assume an MgND profile in response to the increased intraocular pressure, a model of glaucoma.^50^ In the current study, we observed increased microglial expression of *Lgals3* in aged E4 microglia, whereas young E4 microglia had decreased expression relative to E3. These differences in E4 microglia *Lgals3* expression across age and model systems are intriguing, particularly in light of *APOE4* leading to increased risk for AD, yet decreased risk for glaucoma.^50^

KEGG pathway analyses conducted across microglia revealed terms of “Alzheimer’s disease,” “cholesterol metabolism,” and “HIF1α signaling” for the E4-enriched Mi_6 subset. *Hif1α* itself is a DAM gene, and many other important DAM genes (e.g., *Spp1, Igf1*) are also HIF-responsive genes.^17,18,51^ Several recent reports have also demonstrated upregulated HIF1 signaling in AD and collectively point to concurrent activation of both *Hif1α* and OxPhos gene expression as a common feature of amyloid-responding microglia in both humans and mice.^37,52-54^ In line with this, our data also demonstrate that *Hif1α* was a predicted TF enriched in Mi_6. *Hif1α* regulon activity correlated with a cell’s DAM score, a finding that may in part explain the high glycolysis gene expression in this sub-cluster. In addition, in a reduced model system, E4 primary microglia had significantly higher gene expression of *Hif1α* compared with E3 microglia. Further, when we examined microglia harvested from E3 or E4 mice that received systemic LPS administration, the E4-LPS enriched Mi_8 and Mi_11 clusters both showed increased *HIF1α* activity and the highest glycolysis gene expression. Prior reports demonstrate that E4 is consistently tied to increased pro-inflammatory cytokine production after LPS stimulation in both humans^55^ and mice.^56^ Bolstered by our current findings, we propose that this exaggerated inflammatory response, either because of chronic aging or acute pro-inflammatory exposure, may be a consequence of the unique E4-driven metabolic phenotype observed here across multiple paradigms.

Supporting this E4-associated bias toward *Hif1α* activation at the transcriptional level, we observed multiple functional indices of altered metabolism. For example, Seahorse analysis revealed increased aerobic glycolysis and decreased maximal mitochondrial respiration in E4 primary microglia compared with E3. Further, targeted metabolomics showed that E4 microglia display a marked accumulation of succinate and lactate and a decrease in the anti-inflammatory metabolite itaconate. These findings partially align with prior work showing region-dependent alterations in mitochondrial respiration in the E4 mouse brain^30^ and reduced respiration and reduced glycolysis in human induced microglia-like cells (iMGLs) when edited from E3/E3 to E4/E4.^57^ We also note decreased maximal respiration and reliance on glycolysis for ATP production in E4 microglia in response to pro-inflammatory challenge. This may reflect a limit in their ability to engage mitochondrial ATP production, precluding effective tissue repair responses by preventing the switch to an anti-inflammatory phenotype.^11,12^ Our findings also dovetail with recent work in which human E4/E4 iMGLs treated with conditioned media from neuronal spheroids invoked a transcriptional response enriched in HIF-1 signaling and TFs that promote inflammation.^58^ OxPhos gene expression was lower in E4 iMGLs, along with decreased uptake of fatty acids and reduced expression of lipid catabolism genes.^58^ Aside from fatty acids and glucose, it will also be important for future work to consider the relative contributions of other energy substrates in E4 microglia. For example, recent work has highlighted the importance of glutamine as a fuel source for microglia,^59^ and detailed stable-isotope tracing experiments in both mouse and human microglial cell lines have shown that supplementation with the ketone body β-hydroxybutyrate enhances the LPS-induced glycolytic switch and synergistically increased lactate and succinate accumulation.^60^ Collectively, these findings suggest that E4 microglia are predisposed to a pro-glycolytic, pro-inflammatory phenotype, which they are then unable to resolve via metabolic reprogramming, setting up a situation conducive to chronic neuroinflammation.

### A spatial view of *APOE* immunometabolism

We aimed to complement our bulk and scRNA-seq data with a spatially resolved profile of the aging *APOE* transcriptome. ST highlighted the cortex and hippocampus as particularly vulnerable to E4-associated changes, which was exacerbated in the presence of amyloid. Specifically, *APOE4* appeared to exaggerate transcriptional “activation” of microglia and was uniquely associated with alterations in lipid metabolism pathways in plaque-dense microenvironments. Interestingly, Navarro et al.^61^ identified gene expression changes in the hippocampus of 3xTg AD mice that pointed to altered metabolism, including an upregulation of *Lpl* similar to our findings in both Mi_6 of aged E4 mouse brains and the cortex of E4 5XFAD brains by ST. Using App^NL–G-F^ mice, Chen et al.^16^ defined a PIG signature, composed of inflammatory genes induced by close proximity to amyloid plaques, as well as an OLIG-induced gene signature, composed of genes responsible for remyelination. Strikingly, we found that our “microglial activation” module (magenta) strongly overlapped with the PIG signature, being lowest in E3 young, increasing with E4 and age, and highest in E4 5XFAD. This may suggest an exaggerated microglial response to amyloid in E4 brains, in line with previous studies that have demonstrated increased pro-inflammatory responses in E4 microglia.^56,62,63^ Because the PIGs signature is thought to represent dysregulated complement activation,^16^ it is interesting to note that ApoE limits complement activation by forming a complex with C1q, but that the isoforms of ApoE have different binding profiles.^64,65^ Thus, it is conceivable that the PIG^high^/OLIG^low^ profile seen in the E4 brain could lead to increased complement activation, aberrant pruning of synapses, and/or an imbalance in axon myelination, thereby feeding forward into a vicious cycle that propagates neuroinflammation and impaired lipid recycling.

Lipidomic analyses of the postmortem human AD brain have noted changes in brain lipids during the course of the disease.^66^ Recent work extends these findings to include E4-associated decreases in several phospholipid species^67,68^ and isoform-specific microglial responses to ApoE-containing, phospholipid-rich lipoproteins.^69^ Although brain lipidomic profiling typically relies on tissue homogenates from preselected regions, we here leveraged MALDI MSI to simultaneously quantify multiple lipid classes across entire intact brain tissue sections. This allowed us to identify clear regional patterns of lipid expression that were substantially disrupted in the “disease-associated” cortical areas found primarily within the E4 5XFAD brain in our ST analyses. These “disease-associated” brain regions had a gene signature that clearly mapped back to microglia (specifically Mi_6) in our scRNA-seq dataset and was characterized by dramatic upregulation of DAM genes. Further, when we used the ST data to correlate our whole-transcriptome profiles with amyloid plaque intensity on a spot-by-spot basis, we discovered a unique E4 signature that was highlighted by changes almost exclusively in pathways related to lipid metabolism.

Spatially resolved quantification of lipids via MALDI MSI showed clear separation between the overall E3 and E4 brain lipidomes in aged mice and more so in the amyloid-overexpressing background of the 5XFAD brain. In line with our ST findings, they highlight *APOE*- and amyloid-associated decreases in a number of PCs, a class of phospholipid linked to memory decline.^70^ Although a handful of PCs were highest in the E4 5XFAD brain, the majority of PCs were present in lower concentrations relative to the other groups, consistent with findings from the postmortem human brain.^67^ Regional segregation of lipid concentrations revealed that for most PCs, *APOE*-dependent changes in lipid concentrations were more striking in the isocortex, hippocampus, and fiber tracts relative to other brain regions. Strikingly, the top six PCs identified in our clustering analysis, where the highest concentrations were typically seen in the E4 5XFAD brain, are precursors of common phospholipid oxidation products, namely, PCs having arachidonic acid (20:4) and linoleic acid (18:2). The double bonds in these PCs are labile to reactive oxygen species, with their oxidized forms being highly proinflammatory and associated with impaired mitochondrial activity.^71-74^ We also noted *APOE-* and amyloid-associated changes in cholesterol and several triglyceride (TG) species. These findings are intriguing because cholesterol esters and TGs are typically stored in intracellular lipid droplets (LDs), and recent studies have highlighted a role for glial LDs in aging and neurodegeneration, with E4 generally associated with LD accumulation and related metabolic disruptions.^58,75-81^ Together, our bulk, scRNA-seq, ST, and MALDI MSI data suggest that E4 predisposes AD-vulnerable brain regions to neurodegeneration through a metabolism-centered mechanism, perhaps owing to its altered binding profile for lipids and their receptors.^82,83^

Collectively, our data suggest a potential scenario where metabolic dysfunction caused by *APOE4* gives rise to chronic neuroinflammation, linking two phenomena consistently tied to AD with the strongest genetic predictor of the disease. These E4-associated immunometabolic disturbances appear intricately connected to aging and amyloid, with the potential to exacerbate these pathological features and propagate synaptic loss through mechanisms of aberrant microglial activation and lipid dysregulation. This is especially true in the hippocampus and cortex, which were found to be uniquely vulnerable to E4’s immunometabolic reprogramming per our regional analyses. Many potentially modifiable AD risk factors, such as obesity, diabetes, and physical inactivity, also converge on immunometabolic pathways, as do other prominent genetic risk factors, such as *TREM2,*^84^ *CLU*,^85,86^ and *BHLHE40.*^87^ In addition, a recent study identified several WGCNA modules associated with *APOE4* that were enriched for genes involved in lipid and carbohydrate metabolism,^88^ a finding also reflected at the protein level by several large proteomics studies in cortex,^7,89,90^ cerebrospinal fluid (CSF),^7,91,92^ plasma,^93^ and isolated microglia.^94^ Thus, viewing AD through the lens of immunometabolism holds promise to fuse these seemingly disparate risk factors into a comprehensive mechanism whereby impaired microglial metabolism triggers chronic neuroinflammation, sparking the neurodegenerative cascade. Accordingly, therapies that target metabolism and inflammation in tandem may hold greater therapeutic promise in the treatment and prevention of AD.

### Limitations of the study

Our study has several limitations. First, some aspects of AD are not fully captured by mouse models, such as the 5xFAD model used here, which display differences in plaque composition compared with the human brain and lack tau pathology.^95^ However, our use of humanized *APOE* TR (targeted replacement) mice^96-99^ appeared to substantially bridge the gap between human and mouse studies, resulting in high overlap with multiple human datasets. It is also important to point out that although nuclei from neuronal populations are retained well during the snRNA-seq workflow, these cells are more vulnerable to the processing steps of scRNA-seq. Our datasets here are therefore naturally “neuronally depleted” and “glia enriched.” This is beneficial to our ability to survey as many glial cells as possible, but it conversely limits our ability to infer neuronal contributions in the scRNA-seq data. However, neuronal contributions are still represented in our bulk-seq and ST data, which corroborate E4’s role in disrupting immunometabolism. Another limitation is that ST has not yet reached single-cell resolution, such that the cluster differences observed here likely reflect the contributions of multiple cell types. A notable exception in our study was ST cluster 11, which mapped almost exclusively back to microglia cluster Mi_06 in our scRNA-seq data. Related to this, another potential caveat to our study is that differences in plaque load have been reported in the “EFAD” model (E4 > E3), and compared with E3 5XFAD, the E4 5XFAD brain section employed in this study showed a similar increase in plaque+ area as previously reported.^100^ This may confound our ST results by simply exaggerating the microglial response independent of the ApoE isoform present. However, this concern is at least partially mitigated by our spot-by-spot approach, where we controlled for plaque intensity as a variable trait for each x,y coordinate within the spatial transcriptome. In addition, our metabolic analyses in primary microglia are unable to fully model the *in vivo* environment. However, it did allow us to eliminate potential vascular confounds; i.e., because E4 is associated with cerebrovascular dysfunction,^101^ we considered the possibility that local hypoxia and nutrient stress could be driving the increased HIF1α signaling. However, our *in vitro* experiments suggest that increased *HIF1α* and aerobic glycolysis are an innate feature of E4 microglia, because these cells had equal access to nutrients and oxygen in the cell culture medium as their E3 counterparts. Finally, although our findings from scRNA-seq, ST, and MALDI-MSI all point toward an outsized role for E4 in disrupting microglial immunometabolism, the sample sizes in our studies are still limited by the cost-prohibitive and resource-intensive nature of these methods, thus necessitating confirmation.

## STAR★METHODS

### RESOURCE AVAILABILITY

#### Lead contact

Further information and requests for resources and reagents should be directed to and will be fulfilled by the lead contact, Dr. Lance Johnson (johnson.lance@uky.edu).

#### Materials availability

This study did not generate new unique reagents.

#### Data and code availability

- Single-cell RNA-seq, bulk RNA-seq, and spatial transcriptomics data have been deposited at GEO and are publicly available as of the date of publication. MALDI-MSI and GC-MS data have been deposited to Metabolomics Workbench and are publicly available as of the date of publication. Accession numbers are listed in the key resources table. Microscopy data reported in this paper will be shared by the lead contact upon request.
- This paper does not report original code.
- Any additional information required to reanalyze the data reported in this paper is available from the lead contact upon request.

### EXPERIMENTAL MODEL AND SUBJECT DETAILS

#### Human *APOE* mice

Human *APOE* ‘targeted replacement’ (TR) mice homozygous for *APOE3* or *APOE4* were employed across all experiments in the current study (B6.129P2-Apoe^tm2(APOE*3)Mae^ N8, Taconic #1548-F and B6.129P2-Apoe^tm3(APOE*4)Mae^ N8, Taconic #1549-F). In these “knock-in” mice, the coding region (exon 4) of mouse *Apoe* locus was targeted and replaced with the various human *APOE* alleles. Thus, human *APOE* expression remains under control of the endogenous mouse *Apoe* promoter, resulting in a physiologically relevant pattern and level of human ApoE expression.^96-99^ Mice used for the bulk tissue RNAseq and scRNAseq portion of the study were female aged 3 months (young), 12 months (middle aged), or 24 months of age (aged). Mice used in the ST and MALDI MSI experiments were female mice 3 or 24 months of age, or 12 months of age female E3FAD or E4FAD mice (homozygous *APOE* TR mice crossed to the 5XFAD strain^95^ (MMRRC #34840, B6SJL-^Tg(APPSwFlLon, PSEN1*M146L*L286V)6799Vas/Mmjax^)). Mice used for the LPS study were females 12 months of age and intraperitoneally injected with saline or LPS (5 mg/kg bodyweight, from *Escherichia coli* O55:B5 (Sigma #L2880-100MG)) 24 h prior to brain dissection. All mice were group housed in sterile micro-isolator cages (Lab Products, Maywood, NJ), and fed autoclaved food and acidified water ad libitum. Animal protocols were reviewed and approved by the University of Kentucky Institutional Animal Use and Care Committee.

#### Primary cell culture

Primary mixed glial cultures were prepared from postnatal day 0–3 pups of mice homozygous for E3 or E4. The brain was surgically excised and meninges were removed from cortical tissue in ice-cold dissection buffer (Hanks Balanced Salt Solution (Gibco # #14025-076) supplemented with 1% HEPES (Alfa Aesar #A14777), 1M sodium pyruvate (Gibco cat#11360-070), and 1% penicillin/streptomycin (Gibco # 15140-122). After dissection, isolated cortices were stored in a Petri dish on ice containing growth medium (DMEM-F12 (Gibco #11320-033), 10% FBS (VWR# 97068-085), 1% penicillin/streptomycin). Tissue from 4-5 mixed sex pups of the same genotype were pooled. Cortices were finely minced then transferred to a 15mL conical tube and dissociated with 5mL 0.25% trypsin-EDTA (Thermo #25200-056) for 25 min in a 37°C water bath with gentle agitation. An equal amount of growth medium was added to neutralize trypsin and the tubes were centrifuged at 300 x *g* for 5 min. After removing the supernatant, the tissue was washed three times with 2mL of warm HBSS. The tissue was then triturated in 10mL of warm growth medium and passed through a 70μm cell strainer (VWR #10199-657) to remove large particulates. Warm growth medium was added to a final volume of 10mL per mouse brain collected and seeded in T75 flasks (USA Scientific #658-175) incubated at 5% CO_2_ 37°C. Medium was replaced with fresh growth media after 24hr. At 7 DIV medium was replaced with fresh growth medium supplemented with 10% L929 cell-conditioned medium (LCCM, see below for preparation). Peak microglial confluence in the primary mixed glial cultures typically occurred around 12-14 DIV, at which point the flasks were shaken at 240rpm for 2 h at 37°C. Supernatant containing detached microglia was pooled and centrifuged at 300 x *g* for 5 min. Cells were then resuspended and plated in supplemented growth medium incubated at 5% CO_2_ 37°C. All experiments were performed within 2-4 days of plating. Purity of primary microglia cultures was authenticated by immunocytochemistry, with >98% of cells positive for microglia markers P2RY12 (1:400, AnaSpec #AS-55043A) and IBA1 (1:2500, Wako Fujifilm #019-19741). For cytokine stimulation experiments, cells were stimulated with a pro-inflammatory cocktail of 20 ng/ml interferon-γ (IFNγ, R&D Systems #485-MI-100) and 50 ng/ml tumor necrosis factor alpha (TNFα, R&D Systems #410-MT-025).

#### L929 cells

L929 cells (ATCC #CCL-1) are a murine fibroblast cell line that produce growth factors including macrophage colony stimulating factor (M-CSF) that encourage microglial differentiation and proliferation.^112^ The parent L strain was derived from normal subcutaneous areolar and adipose tissue of a 100-day-old male C3H/An mouse. NCTC clone 929 of strain L was derived in March, 1948 by WR Earle and deposited to ATCC. The cell line has been authenticated by ATCC. L929 cells were maintained in DMEM/F12 with 10% FBS and 1% penicillin/streptomycin. In order to prepare L929 cell conditioned medium (LCCM), cell culture supernatant was harvested before passaging every 7 days, at which point it was centrifuged at 300 x *g* for 5 min, sterile filtered through a 0.20μm vacuum filter, and stored at −80C. The same batch of LCCM was used for all primary cultures in this study.

### METHOD DETAILS

#### Seahorse extracellular flux analysis

The Seahorse XF96 Glycolytic Rate Assay (Agilent #103344-100) and Mitochondrial Stress Test (Agilent #103015-100) were performed on E3 and E4 primary microglia to measure glycolysis and mitochondrial respiration, respectively. The Seahorse ATP Rate Assay (Agilent #103592-100) was performed to measure the relative contributions of glycolysis and OxPhos to ATP production. Cells were seeded onto Seahorse XF96 tissue culture microplates (Agilent #101085-004) at a density of 3x10^4^ cells/well in supplemented growth medium (detailed above) and incubated at 5% CO_2_ 37°C. 12 h prior to the start of the assay, cells were stimulated with pro-inflammatory (IFNγ + TNFα) cytokines as described above. The Seahorse Glycolytic Rate Assay (GRA), Mitochondrial Stress Test (MitoStress), and ATP Rate Assay were performed according to manufacturer’s instructions using DMEM-based medium containing 10mM glucose, 2mM glutamine, 1mM pyruvate, pH 7.4. For the GRA, plate was measured under basal conditions followed by serial addition of (A) rotenone and antimycin A (0.5μM) and (B) 2-deoxyglucose (50mM). For MitoStress, plate was measured under basal conditions followed by serial addition of (A) oligomycin (1μM), (B) FCCP (2.0μM), and (C) rotenone and antimycin A (0.5μM). For ATP Rate Assay, plate was measured under basal conditions followed by serial addition of (A) oligomycin (1.5μM) and (B) rotenone and antimycin A (0.5μM). Data were normalized to cell count using the automated Seahorse XF Imaging and Normalization System (Agilent) which utilizes 2μM Hoescht 33,342 (Thermo #62249) to label and count cell nuclei. Data were analyzed using Seahorse Wave v2.6 software (Agilent).

#### Metabolomics

Primary microglia were plated at 7x10^6^ cells/well in 6-well plates (VWR #10062-894) and incubated at 5% CO_2_ 37°C. Upon reaching confluence, cells were removed from the incubator washed with warm 0.9% NaCl solution. Culture plates were placed on a bed of crushed dry ice and 1mL of ice-cold 50% methanol (HPLC-grade, Sigma #A456-4) was added to quench cellular metabolic activity followed by a 10 min incubation at −80°C to ensure cell lysis. After removing from the freezer, cells were detached with a cell scraper (VWR #10062-906) and the entire contents collected into a microcentrifuge tube, vortexed briefly, and placed on ice until all samples were collected. The tubes were then placed on a Disruptor Genie Cell Disruptor Homogenizer (Scientific Industries) for 5 min at 3,000 rpm. Tubes were then centrifuged at 20,000 x g for 10 min at 4°C. The supernatant containing polar metabolites was isolated to a new tube and stored at −80C, and the resulting pellet was briefly dried at 10^−3^ mbar using a CentriVap vacuum concentrator (LabConco) to evaporate remaining methanol, followed by determination of protein content via BCA assay (ThermoFisher #23225) to normalize metabolite concentrations to total protein amount of each sample. The supernatant fraction containing polar metabolites was thawed gently on ice and dried at 10^−3^ mbar followed by derivatization. The dried polar metabolite pellet was derivatized by a two-step methoxyamine protocol first by addition of 70μL methoxyamine HCl (Sigma-Aldrich #226904-5G) in pyridine (20 mg/mL; Sigma-Aldrich #TS25730) to each pellet followed by 90 min dry heat incubation at 30°C. Samples were then centrifuged at 20,000 x g for 10 min after which 50μL of each sample was transferred to an amber V-shaped glass chromatography vial (Agilent #5184-3554) containing 80μL N-methyl-trimethylsilyl-trifluoroacetamide (MSTFA; ThermoFisher #TS48915) and gently vortexed followed by 30 min dry heat incubation at 37^°^C. The samples were allowed to cool to room temperature then analyzed via gas chromatography-mass spectrometry (GCMS). Briefly, a GC temperature gradient of 130°C was held for 4 min, rising at 6 °C/min to 243°C, rising at 60 °C/min to 280°C and held for 2 min. Electron ionization energy was set to 70eV. Scan mode for m/z: 50–550 was used for steady-state metabolomics and scan mode for m/z: 50-800 was used for stable-isotope resolved metabolomics. Spectra were translated to relative abundance using the Automated Mass Spectral Deconvolution and Identification System (AMDIS) v2.73 software with retention time and fragmentation pattern matched to FiehnLib library^113^ with a confidence score of >80. Chromatograms were quantified using Data Extraction for Stable Isotope-labelled metabolites (DExSI) v1.11. Metabolomics data were analyzed using the web-based data processing tool Metaboanalyst.^102^

For stable-isotope resolved metabolomics, cells were washed with warm, sterile phosphate-buffered saline (PBS; Thomas #QZY-11666789001-4L) to remove traces of non-^13^C media and then incubated in glucose- and sodium pyruvate-free DMEM (Thermo #11966-025) containing 2mM GlutaMAX (Thermo #35050-061), 1% penicillin/streptomycin, and 10mM universally labeled ^13^C-glucose (Cambridge Isotope Laboratories # CLM-1396-PK) for 2 h with pro-inflammatory stimulus (IFNγ + TNFα, as described above). After the 2 h incubation, metabolites were extracted from the cells and processed for GCMS as described above. Fractional enrichment was calculated as the relative abundance of each isotopologue relative to the sum of all other isotopologues.

#### Quantitative PCR

E3 and E4 primary microglia were plated at 5x10^6^ cells/well in 6-well plates and RNA was extracted from the cells using the RNEasy Plus Mini Kit (Qiagen #74136) and converted to cDNA using High-Capacity RNA-to-cDNA kit (Thermo #4387406) according to manufacturer’s instructions. TaqMan chemistry was used for quantitative PCR with TaqMan probe targeting *Hif1α* (Thermo #4453320) and TaqMan Fast Advanced Master Mix (Thermo #4444556). PCR was performed on the QuantStudio 3 (Applied Biosystems) with default cycling parameters for this master mix (initial holds at 50°C for 2 min (UNG incubation) and 95°C for 20 s (polymerase activation) then 40 cycles of denaturation at 95°C for 1 s followed by annealing/extension at 60°C for 20 s). Data were analyzed using the ddCT method with 18s ribosomal rRNA (TaqMan assay id# Hs99999901_s1) as the reference gene.

#### Brain single-cell suspension, cDNA library, and sequencing

Pooled brain tissue (3 biological replicates per experimental group)) was processed for ‘glia-enriched’ single cell suspensions.^114^ Three different mice for each experimental group (18 mice in total for Figure 1 and 2; 12 mice total for Figure 3) were anesthetized via 5.0% isoflurane before exsanguination and transcardial perfusion with ice-cold Dulbecco’s phosphate buffered saline (DPBS; Gibco # 14040133). Following perfusion, brains were quickly removed, the three biological replicates were pooled in a single Petri dish, and whole left hemispheres sans brainstem and cerebellum were quickly minced using forceps on top of an ice-chilled Petri dish. Minced tissue from the 3 pooled hemispheres per group were immediately transferred into gentleMACS C-tube (Miltenyi #130–093-237) containing Adult Brain Dissociation Kit (ADBK) enzymatic digest reagents (Miltenyi #130–107-677) prepared according to manufacturer’s protocol. Tissues were dissociated using the “37C_ABDK” protocol on the gentleMACS Octo Dissociator instrument (Miltenyi #130–095-937) with heaters attached. After tissue digestion, cell suspensions were filtered through 70 μm mesh cell filters to remove debris following the manufacturer’s suggested ABDK protocol. The resultant suspension was sequentially filtered (x2) using fresh 30 μm mesh filters. Cell viability was checked using AO/PI viability kit (Logos Biosystems # LGBD10012). All cell suspensions were determined to have >90% viable cells. Following viability and counting, cells were diluted to achieve a concentration of ~1700 cells/μL in a 10μL total reaction volume. The diluted cell suspensions were loaded onto the 10X Chromium Connect automated cell portioning system. Sample libraries were constructed using Next GEM automated 3′ reagents (10X Genomics, v3.1) following manufacturer’s suggested protocol (#CG000286 Rev B). Final library quantification and quality check was performed using BioAnalyzer (Agilent), and sequencing performed on a NovaSeq 6000 S4 flow cell, 150 bp Paired-End sequencing (Novogene).

#### scRNAseq data processing

After libraries were sequenced and quality control was performed, samples were aligned to the mm10 mouse reference genome using the Cell Ranger 6.0.2 pipeline. Each sample was aggregated using the cellranger aggr function to produce a raw UMI count matrix containing the number of reads for genes in each cell per sample. The expression matrix was loaded into R for further analysis and visualization using Seurat (v.4.1.0)^105^. Cells were then filtered to reduce the potential of including doublet and low-quality cells using the following criteria: 200 < nGene <5000; 500 < nCount <90,000; and percent.mito <30%. Feature counts were normalized using LogNormalize method with a scale factor of 10,000 (default option); and the effects of percent.mito were regressed out using the ScaleData method. A shared nearest neighbor (SNN) graph was constructed using FindNeighbors function with default parameters. Using the Louvain algorithm implemented in FindClusters function and the first 15 principal components (PCs), we identified 34 unique clusters.

To assign glial cell type identity to each cluster, we manually examined the expression levels of cell type-specific markers across each cluster using Partek Flow software (Partek) to identify clusters containing unique populations of different cell types. Canonical CNS cell type markers were compiled from^115-118^ and included: *Aldoc, Aqp4, Gja1, Aldh1l1, Gfap, Slc7a10, Sox9* (Astrocytes), *P2ry12, Tmem119, Aif1, Slc2a5, Trem2, Cx3cr1, Itgam, Gpr34, C3ar1, Csf1r, Fcrls* (Microglia), *Mgl2, Mrc1, Pf4* (Macrophages), *Mog, Opalin, Mag, Ermn, Cldn11* (Oligodendrocytes), *Pdgfra, Opcml, Tnr, Myt1* (Oligodendrocyte precursors), *Kl, Car12, Ttr* (Choroid plexus), *Ccdc153, Dnah11* (Ependymal), *Cd3d* (Lymphocytes), *Flt1, Emcn, Cldn5, Cdh5, Vwf, Tek, Cd34* (Endothelial), *Slc47a1, Mgp* (Vascular leptomeningeal), *Acta2, Bgn* (Vascular smooth muscle), *Vtn, Kcnj8* (Pericytes), and *Dcx* (Neuroprogenitors). This process resulted in stringent filtering of cells with ambiguous assignments (>1 cell-specific gene marker; likely ‘doublet’ and ‘triplet’ that slipped through the 10X ‘single cell’ droplet workflow), leaving a total of 39,475 cells within 24 carefully assigned glial clusters.

#### Re-clustering of specific glial cell populations (ex. microglia)

We used the FindAllMarkers function to identify genes that act as markers for each cluster, using the Wilcoxon rank-sum test. A gene was considered the marker of a cluster if it had a Bonferroni-adjusted p value <0.01 and an average log fold change >0.1. The data were further filtered to contain only astrocytes, microglia or other glial cell types using the markers described above. After re-clustering with a resolution of 0.1 and the first 15 PCs, we identified 11 microglia sub-clusters and 12 astrocyte sub-clusters. To perform differential expression analysis in the cell-specific datasets, we used Seurat’s FindMarkers function and performed Wilcoxon rank-sum tests. A gene was considered differentially expressed if it had a Bonferroni-corrected p value <0.05 and a natural log fold change (logFC) > 0.25.

#### Pathway enrichment analyses

The Seurat function FindMarkers conducted the DEG analysis via grouping for comparison by clusters, *APOE* genotype, or age (min.pct was set as 0.25 and logFC.threshold was set as 0.25). The DEGs were selected if the adjusted p value was less than 0.05 and the absolute value of log-fold change was higher than 0.1. The KEGG enrichment analyses described in Figure 1 were performed using Partek Flow’s GSA DE feature. Based on the identified DEGs, the enrichment analyses of GO terms (Biological Process (BP) were performed via Enrichr^106^ or the rWikiPathways R software package^107^ with cutoff by FDR-adjusted adjusted p values 0.05. The bar-plot functions from the software package with a color-blind-friendly color scheme were applied for the visualizations.

#### Gene set enrichment analysis for metabolic pathways gene signatures

AUCell R software package (v.1.14.0) was applied for the identification of gene signatures at the single-cell level.^34^ AUCell uses the "Area Under the Curve" (AUC) to calculate whether a critical subset of the input gene set is enriched within the expressed genes for each cell. AUC scores were calculated for each individual cell and distribution across cell populations of interest allowed for exploration of the relative expression of the gene signature. AUCell scores of seventy KEGG pathway gene sets associated with pathways of mammalian metabolism (https://www.genome.jp/kegg/pathway.html) were manually curated and applied to multiple datasets. All pathways organized under the KEGG umbrella term “Metabolism” were considered metabolic pathways, however non-mammalian pathways were removed (ex. “Photosynthesis”) and multiple sub-pathways were condensed into more manageable lists. For example, our pathway “Glycosaminoglycan biosynthesis” contains lists for three small sub-pathways “-chondroitin sulfate/dermatan sulfate”, “heparan sulfate/heparin”, and “keratan sulfate”. This curation, based off of previous publications and designed to infer maximal biological relevance,^119^ resulted in a total of 79 metabolic pathways. Each pathway and its corresponding gene lists are detailed in Table 3. Finally, the average AUCell scores of each KEGG metabolic pathway were plotted as heatmaps using pheatmap R software (v.1.0.12) sorted by either cell subtypes and/or experimental groups.

#### Gene-gene network analyses using WGCNA

Weighted gene co-expression network analysis (WGCNA) (v1.70-3)^108^ was used to identify gene modules and build unsigned co-expression networks, including both negative and positive correlations. Briefly, WGCNA constructs a gene co-expression matrix, uses hierarchical clustering in combination with the Pearson correlation coefficient to cluster genes into groups of closely co-expressed genes termed modules, and then uses singular value decomposition (SVD) values as module eigengenes (MEs) to determine the similarity between gene modules or calculate the association between module and a preselected sample trait (ex. *APOE* genotype, treatment, or plaque intensity). For both the spatial transcriptomics WCGNA and plaque intensity correlation analyses (Figure 6) we include all spots from the two 5xFAD samples (E3 5XFAD and E4 5XFAD). For each analysis, the top 3,000 variable genes were selected to identify gene modules and network construction. Soft power of 6 was chosen by the WGCNA function pickSoftThreshold. Next the function TOMsimilarityFromExpr was used to calculate the TOM (Topological Overlap Matrix) similarity matrix via setting power = 6, networkType = "signed”. The distance matrix was generated by subtracting the values from the similarity adjacency matrix by one. The function flashClust (v.1.01) was used to cluster genes based on the distance matrix, and the function cutreeDynamic was utilized to identify gene modules by setting deepSplit = 3. Cytoscape (v.3.8.2) was applied for the gene-gene network visualization.

#### Gene score plots

Pairwise differential expression analyses were then performed between E4 vs E3, aged vs young, aged vs middle, and middle vs young. For each gene within each differential expression analysis, a gene score was calculated to represent a combination of effect size and statistical significance of the differential expression. The gene score was calculated as the product of the log2 fold change (FC) and negative of the log-transformed false discovery rate (FDR), log2(FC)*-log10(FDR).

#### Gene transcriptional regulatory network analyses using pySCENIC

For regulon identification, gene regulatory network analysis was performed using the pySCENIC software packages (v.0.11.2)^110^. The arboreto package is used for this step using the algorithm of GRNBoost2 (version 0.11.2) to identify the potential transcriptional factor (TF)-targets based on their co-expression with RcisTarget (version 1.12.0) for cis-regulatory motif enrichment analysis in the promoter of target genes (mm9-500bp-upstream-10species.mc9nr and mm9-tss-centered-10kb-10species.mc9nr databases provided in the pySCENIC package), and to identify the regulon, which consists of a TF and its co-expressed target genes. Correlations between a list of 1,390 human transcription factors (TFs) curated by Lambert et al.^120^ and the genes in the expression matrix were evaluated, and co-expression modules with a minimum size of 20 genes were defined. Finally, for each regulon, pySCENIC uses the AUCell algorithm to score the regulon activity in each cell. The input for SCENIC was the n (genes) by n (cells) matrix obtained after filtering, and gene expression is reported in count units. Parameters used for running were specified as default options in the original pySCENIC pipeline. The cellular activity pattern of a predicted regulon can be binarized as being in an ‘on’ or ‘off’ state based on the bimodal distribution of a regulon’s AUCell values and visualized as a heatmap for identification of regulon clustering.

#### Brain preparation for spatial transcriptomics

The mirroring hemisphere (right) from brains processed for scRNAseq (see STAR Methods section “brain single-cell suspension, cDNA library, and sequencing”) were immediately placed in OCT compound (Fisher HealthCare Tissue Plus O.C.T. Compound Clear 4585) and gently lowered into isopentane (Sigma-Aldrich 2-Methylbutane M32631) in a beaker surrounded by dry ice (isopentane chilled to approximately −70°C). Brains were submerged for 60 s, placed on dry ice, wrapped in aluminum foil, and stored at −80°C until sectioning. Prepared brain hemispheres were cryosectioned to 10 μm thick coronal sections at approximately bregma −2.00 mm. Serial 10 μm sections immediately rostral and caudal to the section mounted on the Visium Spatial Gene Expression slide (10X Genomics) were collected for immunohistochemistry. Optimal tissue permeabilization time was determined using the manufacturer’s optimization protocols (10X Genomics, Visium Spatial Tissue Optimization), and accordingly, experimental tissues were permeabilized for 18 min for Visium Spatial Gene Expression analysis. Prior to library preparation, tissue sections were methanol-fixed, stained with hematoxylin and eosin (H&E) (VWR 95057-844), and imaged on a Nikon NiU microscope with Fi3 color camera. Sections were then permeabilized and processed to obtain cDNA libraries, which were subsequently prepared according to the manufacturer’s protocol (https://support.10xgenomics.com/spatial-gene-expression/library-prep). Final library quantification and quality check was performed using BioAnalyzer (Agilent), and sequencing performed on a NovaSeq 6000 S4 flow cell, 150 bp Paired-End sequencing (Novogene).

#### Spatial transcriptomics data processing

Raw FASTQ data and H&E images were processed by the Space Ranger v1.3.0 (10X Genomics) pipeline. Illumina base call (BCL) files from the sequencing instrument were converted to FASTQ format for each sample using the mkfastq. Visium spatial expression libraries were analyzed with the count command. Image alignment to predefined spots was performed by the fiducial alignment grid of the tissue image to determine the orientation and position of the input image. Sequencing reads were aligned to the mm10 reference genome using STAR (v2.5.1b) aligner. Gene expression profiling in each spot was performed with UMI and 10X barcode information. The spots with gene expression data were analyzed with the Seurat package (v.4.1.0). Gene counts were normalized using ‘Log-Normalize’ methods in Seurat. The top highly variable genes (n = 3,000) were then identified using the ‘vst’ method in Seurat. The number of RNA counts for each spot and the frequency of mitochondrial gene counts were regressed out in the scaling process. Six spatial transcriptomic datasets were merged and rescaled (E3 young, E4 young, E3 aged, E4 aged, E3 5XFAD, E4 5XFAD). Principal component analysis was performed using the top highly variable genes. For visualization, dimension reduction was performed using UMAP on the top 20 principal components were applied. Graph-based clustering based on the Louvain community detection algorithm was performed. Markers for each cluster were identified by Wilcoxon rank-sum tests for a given cluster vs. other clusters implemented in Seurat as a ‘FindAllMarkers’ function.

#### Integrative analysis of amyloid plaque intensity and spatial transcriptomic data

The anatomical location of each cluster was visually identified by comparison with the Allen Mouse Brain Reference Atlas (https://mouse.brain-map.org/static/atlas). The region annotation information (ex. isocortex, fiber tracts, etc.) was integrated as spot metadata. Separately, the amyloid plaque (X-34 stained) images were prepared from a 10 μM section immediately adjacent (caudal) to the 10 μM section used for the ST data generation. The plaque image was resized to exactly match the same-section H&E image for the ST coordinates. Cropping and rotation were performed to overlap both images, and the color channels specifically addressing the plaque intensities (X-34, blue) were extracted using the Photoshop image analysis tool. The quantitative extraction of plaque intensity scores was performed using the Squidpy software package (version 1.0.0)^111^. The resulting plaque intensity score values were added as spot metadata for downstream analyses.

#### Ligand–receptor cell-cell interactions

Cell-to-cell communication was identified by evaluating the expression of pairs of ligands and receptors within cell populations using the CellChat R software package (version 1.1.3)^109^. CellChat infers the biologically significant cell-cell communication by assigning each interaction with a probability value and performing a permutation test. CellChat models the probability of cell-cell communication by integrating gene expression with prior known knowledge of the interactions between signaling ligands, receptors, and their cofactors using the law of mass action. We examined the interaction among different cell types or microglia and astrocyte subtypes. The databases, including ‘Secreted Signaling’ provided by Cellchat, were used. To identify the ligand-receptor interactions of ApoE, a gene list of *APOE* and target receptors (ex. *Ldlr*) reported by Sheikh et al.^121^ was added to the receptor-ligand interaction database of CellChat. The ApoE-ApoE receptor list is included in Table 1.

#### Matrix assisted laser desorption ionization (MALDI) mass spectrometry imaging (MSI)

Brain sections (10 μm) were mounted on glass slides and prepared for MALDI MSI (see STAR Methods section “brain preparation for spatial transcriptomics“). Slides were prepared as follows.^122^ After desiccation for 1 h, slides were sprayed with 14 passes of 7 mg/mL N-(1-Naphthyl) ethylenediamine dihydrochloride (NEDC) matrix (Sigma) in 70% methanol (HPLC-grade, Sigma) was applied at 0.06 mL/min with a 3mm offset and a velocity of 1200 mm/min at 30°C and 10psi using the M5 Sprayer with a heated tray of 50°C. Slides were used immediately or stored in a desiccator until analysis. For the detection of lipids, a Waters SynaptG2-Xs high-definition mass spectrometer equipped with traveling wave ion mobility was employed with the flellowing parameters.^122^ The laser was operating at 2000 Hz with an energy of 300 AU and spot size of 50 μm at X and Y coordinates of 100μm with mass range set at 50–1000 *m/z* in negative mode. MALDI-MSI data files were processed to adjust for mass drift during the MALDI scan and to enhance image quality and improve signal-to-noise ratio using an algorithm available within the High-Definition Imaging (HDI) software (Waters Corp). To adjust for mass drift during the MALDI scan, raw files were processed using a carefully curated list of 20 MALDI NEDC matrix peaks (*m/z*) 26 small molecule MALDI peaks(*m/z*), and 24 lipid peaks(*m/z*) validated by spotting standards. Files were processed at a sample duration of 10 s at a frequency rate of 0.5 min, and an *m/z* window of 0.1 Da, using an internal lock mass of previously defined metabolite of taurine 124.007 *m/z* with a tolerance of 1amu and a minimum signal intensity of 100,000 counts. Data acquisition spectrums were uploaded to the HDI software for the generation of lipid images. Regions of interest (ROIs) were user defined by a blinded investigator using anatomical reference points based on the mouse Allen Brain atlas. For all pixels defined within a ROI, peak intensities were averaged and normalized by total ion current (TIC) and number of pixels.

#### Immunohistochemistry

Brains were sectioned coronally at 10 μm at approximately bregma −2.00 mm. Serial 10 μm sections immediately rostral and caudal to the section mounted on the Visium Spatial Gene Expression slide (10X Genomics) were collected for immunohistochemistry and stored in cryoprotectant at −20 °C. Primary and secondary antibodies were diluted in 3% normal goat serum (LAMPIRE Biological Laboratories #7332500) with 0.2% Triton X-100 (Sigma CAS #9036-19-5). The tissue was blocked in 10% normal goat serum with 0.2% Triton X-100. Sections were incubated overnight at 4 °C with rabbit anti-P2ry12 (Anaspec #AS-55043A, 1:400), rat anti-GFAP (Invitrogen #13-0300, 1:400), followed by PBS wash and incubation with goat anti-rat AF568 (Invitrogen #A11077, 1:400) and goat anti-rabbit AF488 (Invitrogen #A11304, 1:200) for 2 h at room temperature. The sections were then washed, mounted on slides, and allowed to dry overnight. The slides mounted with dried tissue were then incubated in X-34 (Sigma #SML1954) 10μg/mL solution for 10 min at room temperature before being washed in PBS and differentiated in 80% ethanol for 1 min. The slides were coverslipped with ProLong Gold Antifade Mountant (ThermoFisher #P10144) and imaged on a Zeiss Axio Scan Z1 digital slide scanner at 20× magnification.

### QUANTIFICATION AND STATISTICAL ANALYSIS

For the bulk seq data in Figures 1D and S2A, n = 3-5, *APOE* and Age effects were analyzed using a two-way ANOVA, and significance was noted as p < 0.05. Error bars represent standard error. MALDI-MSI data were analyzed using multiple comparisons ANOVA in Prism v9.0 software (GraphPad). Statistical significance for gene list overlap was determined using a hypergeometric distribution test using the dhyper and phyper functions in R. For *in vitro* primary microglia experiments (Figure 4), cells from each litter of mice are pooled together before final replating for experiments. Therefore, each *n* represents an individual well in the final cell culture vessel used for experiment. For targeted metabolomics and qRT-PCR, each *n* is one well of a 6-well plate (n = 6 per group for qRT-PCR, n = 21-22 per group for metabolomics). Metabolomics data are combined results from four independent GCMS runs from different batches of primary microglia. For metabolomics data, two-tailed T tests adjusted for multiple comparisons were performed using Metaboanalyst v5.0^102^. For qRT-PCR, samples were run in triplicates and statistical significance assessed using a two-tailed T test in Prism software (v9.0, GraphPad). For Seahorse experiments, each *n* represents one well of the Seahorse 96-well plate. Data shown for Glycolytic Rate Assay (Figures 4E and 4F) and the MitoStress assay (Figures S5C-S5F) are representative of three independent assays for each, with n = 15-16 per group per assay for the GRA and n = 12-15 per group per assay for MitoStress. Data shown for the ATP Rate Assay represent data from a single assay with n = 5-9 per group (Figures 4G and 4H). Statistical significance was assessed using two-tailed T tests (GRA, MitoStress) or two-way ANOVA with Tukey post-hoc test for multiple comparisons (ATP Rate Assay) in Prism software (v9.0, GraphPad).

## Supplementary Material

1

2

3

4

## Figures and Tables

**Table:** KEY RESOURCES TABLE

REAGENT or RESOURCE	SOURCE	IDENTIFIER
Antibodies		
P2RY12	AnaSpec	AnaSpec; EGT Group Cat# 55043A, RRID:AB_2298886
GFAP	Invitrogen	Thermo Fisher Scientific Cat# 13-0300, RRID:AB_2532994
IBA1	Wako Fujifilm	FUJIFILM Wako Shibayagi Cat# 019-19741, RRID:AB_839504
Chemicals, peptides, and recombinant proteins		
Recombinant Mouse IFN-gamma Protein	R&D Biosystems	485-MI-100
Recombinant Mouse TNF-alpha (aa 80-235) Protein	R&D Biosystems	410-MT-025
Lipopolysaccharides from Escherichia coli O55:B5	Sigma Aldrich	L2880-100MG
X-34	Sigma Aldrich	SML1954
Critical commercial assays		
Seahorse XF Glycolytic Rate Assay	Agilent	103344-100
Seahorse XF Mitochondrial Stress Test	Agilent	103015-100
Seahorse XF ATP Rate Assay	Agilent	101085-004
RNEasy Plus Mini Kit	Qiagen	74136
High Capacity RNA-to-cDNA Kit	Thermo	4387406
Adult Brain Dissociation Kit, mouse and rat	Miltenyi	130–107-677
Acridine Orange / Propidium Iodide Cell Viability Kit	Logos Biosystems	LGBD10012
Deposited data		
Bulk RNA-sequencing, APOE x aging	This paper (Figure 1)	GEO: GSE212343
Single-cell RNA-sequencing, APOE x aging	This paper (Figure 1 and 2)	GEO: GSE212317
Single cell RNA-sequencing, APOE x LPS	This paper (Figure 3)	GEO: GSE215444
Spatial transcriptomics, APOE x aging and amyloid	This paper (Figure 5 and 6)	GEO: GSE212323
Primary microglia metabolomics, GC-MS, Steady state	This paper (Figure 4)	Metabolomics Workbench: PR000639
Primary microglia metabolomics, GC-MS, 13c Glucose SIRM	This paper (Figure 4)	Metabolomics Workbench: PR000639
APOE x Amyloid, MALDI-MSI	This paper (Figure 7)	Metabolomics Workbench: PR000639
Experimental models: Cell lines		
L929 fibroblasts	ATCC	#CCL-1
Experimental models: Organisms/strains		
Mouse: B6SJL-Tg(APPSwFlLon, PSEN1*M146L*L286V)6799Vas/Mmjax	The Jackson Laboratory	MMRRC stock #34840; RRID: MMRRC_034840-JAX
Mouse: B6.129P2-*Apoe*^tm3(APOE*4)Mae^N8	Taconic Biosciences	Model #1549-F
Mouse: B6.129P2-*Apoe*^tm2(APOE*3)Mae^N8	Taconic Biosciences	Model #1548-F
Oligonucleotides		
*Hif1a* Taqman Gene Expression Assay	Thermo	Taqman Assay ID# 4453320
18s rRNA Taqman Gene Expression Assay	Thermo	Taqman Assay ID# Hs99999901_s1
Software and algorithms		
MetaboAnalyst v5.0	(Pang et al)^102^	www.metaboanalyst.ca
Seahorse Wave v2.6	Agilent	https://www.agilent.com/en/product/cell-analysis/real-time-cell-metabolic-analysis/xf-software/seahorse-wave-desktop-software-740897
Prism v9.0	GraphPad	www.graphpad.com
Automated Mass Spectral Deconvolution and Identification System (AMDIS) v2.73	(Davies et al)^103^	www.amdis.net
Data Extraction for Stable Isotope-labelled metabolites (DExSI) v1.11	(Dagley et al)^104^	https://doi.org/10.1093/bioinformatics/bty025
Cell Ranger v6.0.2	10X Genomics	https://support.10xgenomics.com/single-cell-gene-expression/software/downloads/latest
Space Ranger v1.3.0	10X Genomics	https://support.10xgenomics.com/spatial-gene-expression/software/pipelines/latest/installation
Seurat v4.1.0	(Hao et al)^105^	https://doi.org/10.1016/j.cell.2021.04.048
Partek Flow Software	Partek	https://www.partek.com/partek-flow/
Enrichr	(Kuleshov et al)^106^	https://doi.org/10.1093/nar/gkw377
WikiPathways	(Slenter et al)^107^	https://doi.org/10.1093/nar/gkx1064
WCGNA v1.70-3	(Langfelder et al)^108^	https://doi.org/10.1186/1471-2105-9-559
CellChat v1.1.3	(Jin et al)^109^	https://doi.org/10.1038/s41467-021-21246-9
AUCell v1.14.0	(Aibar et al)^34^	https://doi.org/10.1038/nmeth.4463
pySCENIC v0.11.2	(van de Sande et al)^110^	https://doi.org/10.1038/s41596-020-0336-2
Squidpy v1.0.0	(Palla et al)^111^	https://doi.org/10.1038/s41592-021-01358-2

## Highlights

- *APOE4* and age interact to drive DAM-like signatures in the absence of AD pathology
- APOE4 microglia have increased aerobic glycolysis and higher *Hif1α* expression
- APOE4 exacerbates plaque-induced microglial reactivity and lipid metabolism
- Mass spectrometry imaging reveals distinct phospholipid distribution in E4FAD brains

## INCLUSION AND DIVERSITY

One or more of the authors of this paper self-identifies as an underrepresented ethnic minority in their field of research or within their geographical location. One or more of the authors of this paper self-identifies as a gender minority in their field of research. One or more of the authors of this paper self-identifies as a member of the LGBTQIA+ community. One or more of the authors of this paper received support from a program designed to increase minority representation in their field of research.
