# Supplementary material for: APOE modulates microglial immunometabolism in response to age, amyloid pathology, and inflammatory challenge
Source: Cell Rep. Author manuscript; Available in PMC 2023 Apr 20. (PMC10117631; doi:10.1016/j.celrep.2023.112196)
Supplement: 1 [file NIHMS1887279-supplement-1.pdf]

Cell Reports, Volume *42*

Supplemental information

APOE modulates microglial immunometabolism in response to age, amyloid pathology, and inflammatory challenge

Sangderk Lee, Nicholas A. Devanney, Lesley R. Golden, Cathryn T. Smith, James L. Schwartz, Adeline E. Walsh, Harrison A. Clarke, Danielle S. Goulding, Elizabeth J. Allenger, Gabriella Morillo-Segovia, Cassi M. Friday, Amy A. Gorman, Tara R. Hawkinson, Steven M. MacLean, Holden C. Williams, Ramon C. Sun, Josh M. Morganti, and Lance A. Johnson

Supplemental Figures and Figure Legends

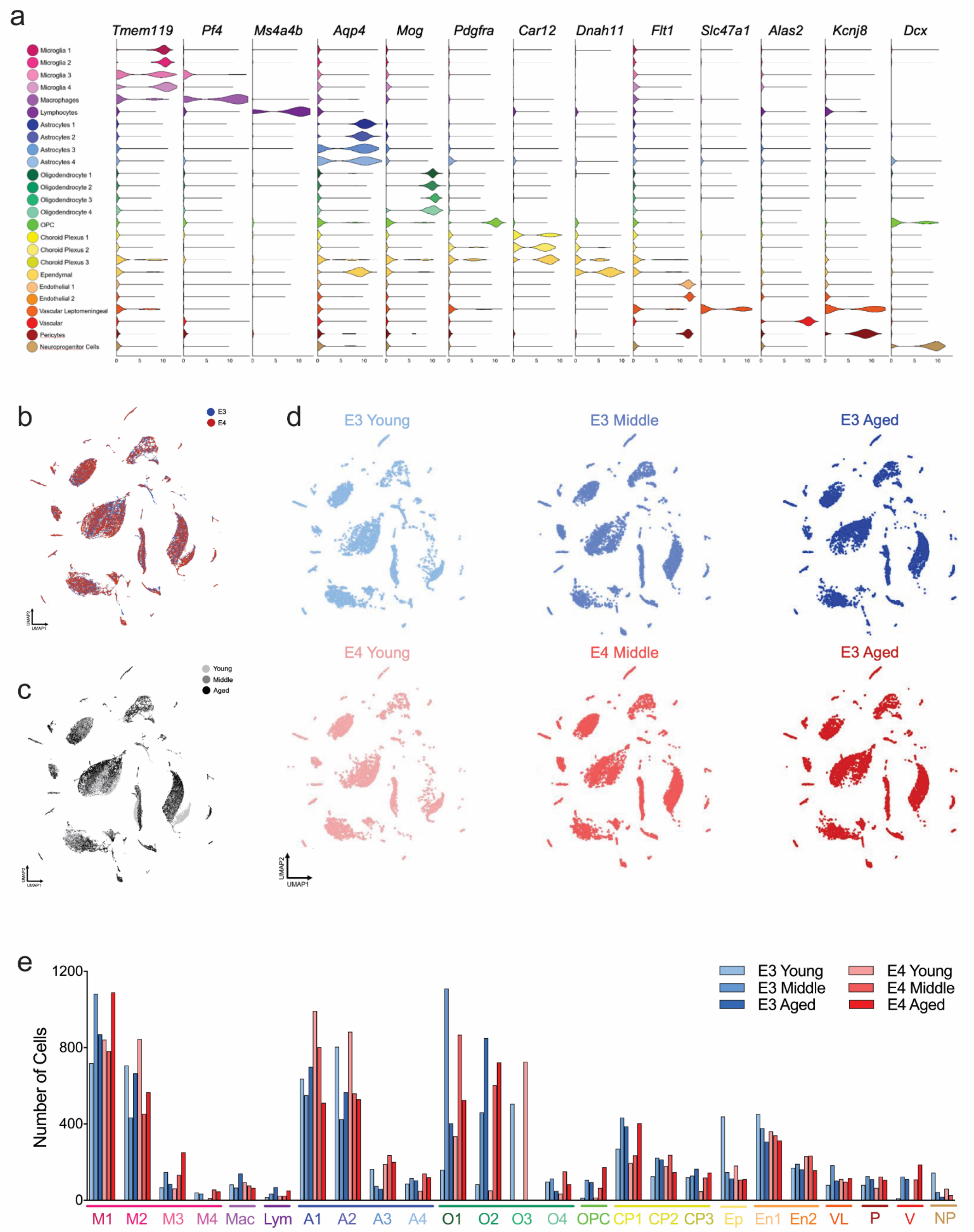

Fig. S1 (related to Fig 1). Cell-specific markers, UMAPs and cell numbers per cluster. a) Gene expression of cell-specific markers for each cluster. b-d) UMAP plots of all cells color coded by *APOE* genotype (b) or age (c), or split by experimental group (d). e) Total number of cells in each cluster.

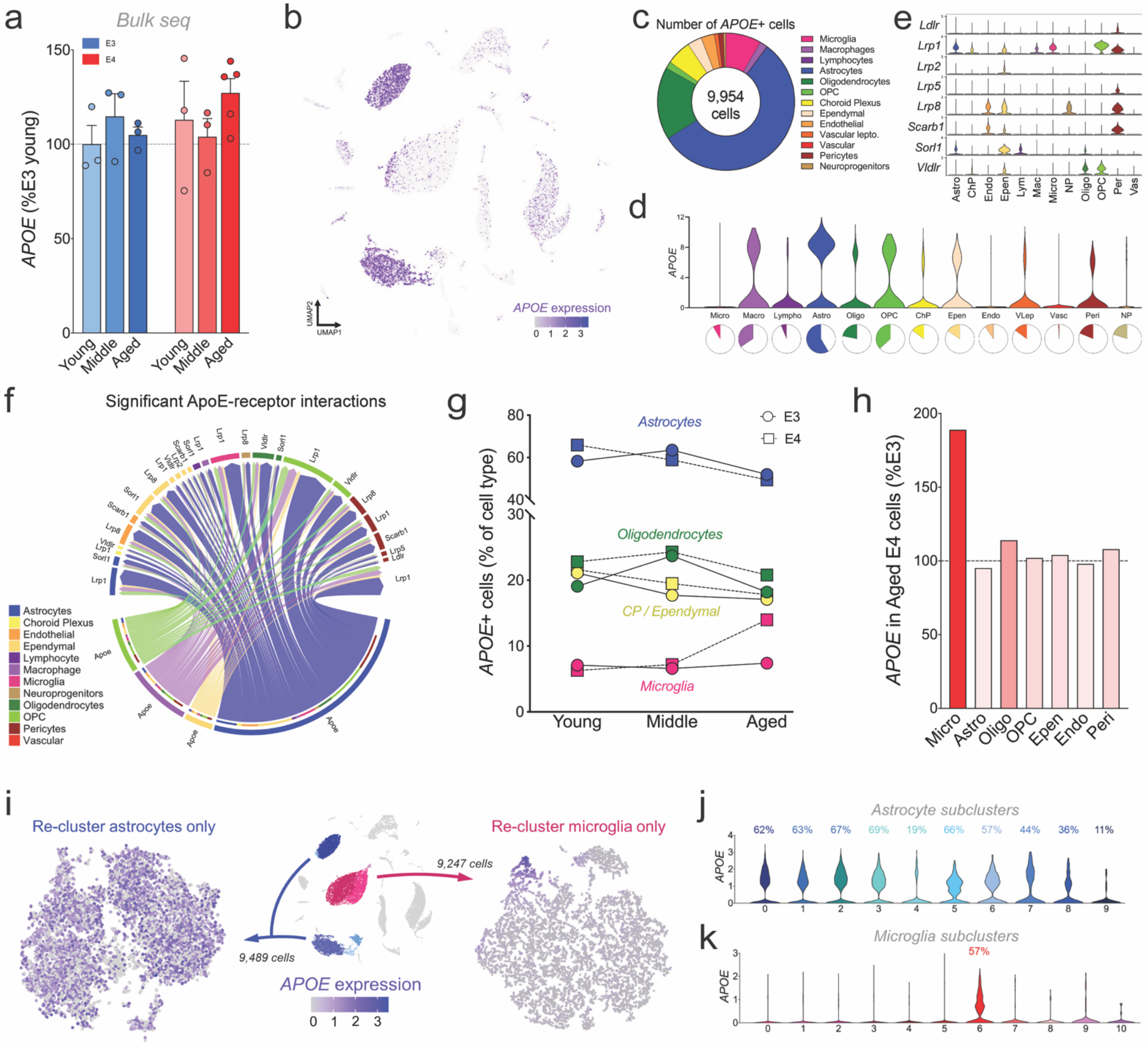

Fig. S2 (related to Fig 1). *APOE* expression is selectively upregulated in aged E4 microglia. a) *APOE* expression in whole brain tissue across the lifespan, relative to *APOE* expression in E3 young. ('Bulk seq'). E3 shown in blue, E4 shown in red. p=0.39 for *APOE*, p=0.68 for age as determined by 2-way ANOVA. n=3-5. b) UMAP showing *APOE* expression across all cell types (scRNAseq). c-e) Donut chart of all 9,954 cells expressing *APOE* at a detectable level ("*APOE*+"). Color slices denote the fraction of total *APOE*+ cells belonging to each cell type. d) Violin plots showing *APOE* expression across each cell type. Pie charts (bottom) show the percent of each cell type that are *APOE*+. e) Expression of established ApoE receptors within each glial cell type. f) Circos plot showing significant ApoE-receptor interactions as calculated by CellChat. Astrocytes (bottom, blue) express the majority of *APOE* and signal to a variety of ApoE receptors across multiple cell types (top). Bottom: outer circle color denotes the cell type expressing *APOE,* while the inner circle color denotes the cell type expressing the corresponding receptor (ex. *Ldlr*). g) *APOE* expression 'over time' in the primary *APOE* expressing cell types (from c). The percent of *APOE*+ cells within each glial cell type is shown in young, middle aged, and aged E3 and E4 brains. h) *APOE* expression in aged E4 glia as a percent of aged E3 cells for each cell type. Expression of *APOE* is selectively upregulated in aged E4 microglia. i-k) *APOE* expression is restricted to a subset of microglia. i) tSNE plots of astrocytes (left) and microglia (right) across all ages, showing expression of *APOE* within each cell type. *APOE* is highly expressed across the majority of astrocyte sub-clusters (j), while expression in microglia (k) is restricted to Mi_ 6.

`

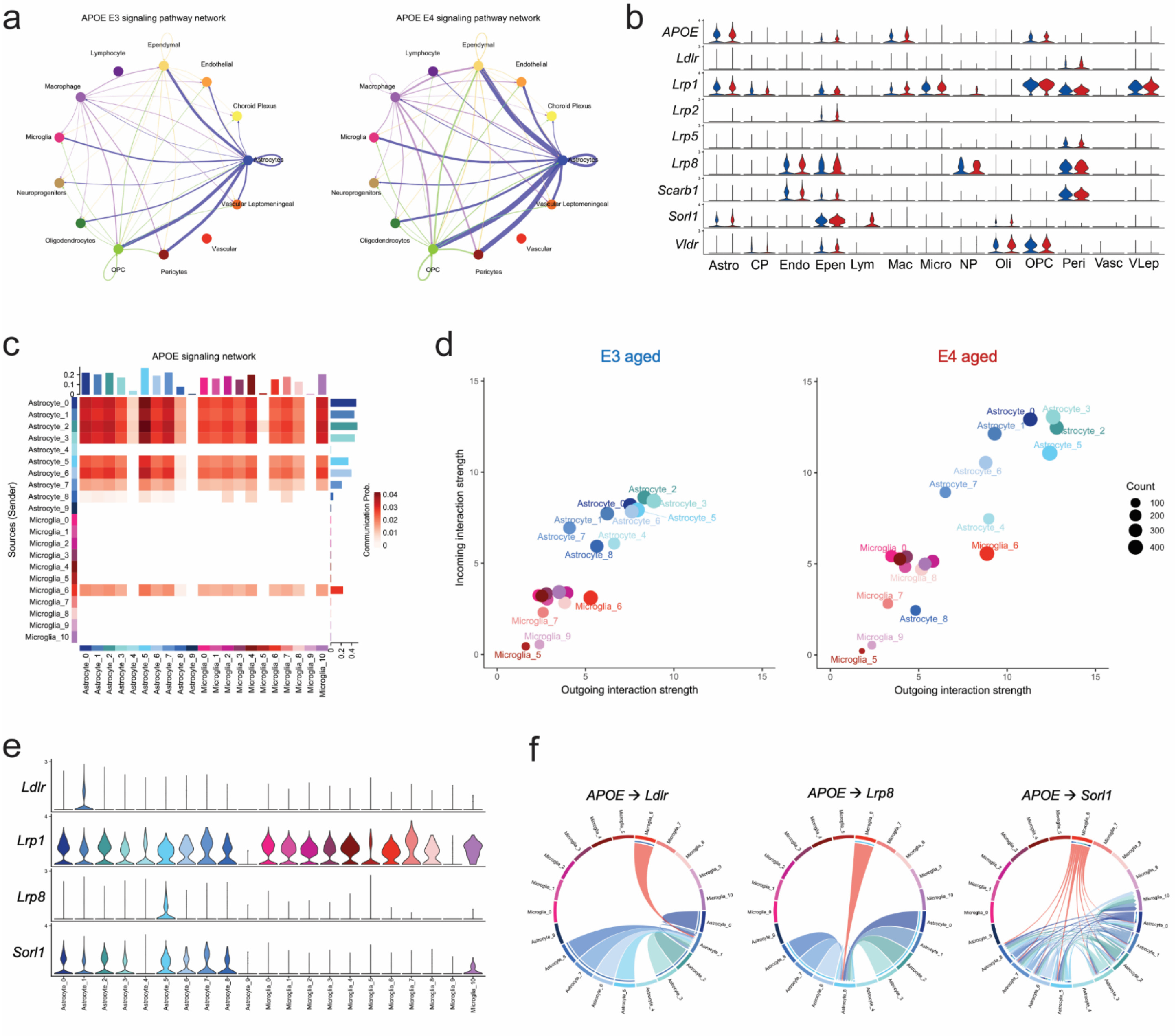

Fig. S3 (related to Fig. 1). ApoE-ApoE receptor signaling pathway network analyses. a) Circle plot of signaling pathway networks for E3 (left) and E4 (right) cell types. Lines connecting cell types denote the aggregated cell-cell communication probabilities evaluated from the expression of the receptors, ligands, and cofactors in different types in the scRNA-Seq dataset. The width of lines corresponds to the signaling pathways' relative interaction strength (aggregated communication probabilities). b) Expression of *APOE* and various ApoE receptors across all glial cell types in E3 (blue) and E4 (red). c) Heatmap of the ApoE-ApoE receptor signaling pathway network across the various astrocyte and microglia subclusters in the E3 and E4 cell datasets. Heatmap color represents communication probability, i.e. the probability of cell-cell communication by integrating gene expression with prior known knowledge of the interactions between signaling ligands, receptors, and their cofactors. Bars represent the sum of the communication probabilities of each subtype. X- and Y-axis indicate the targets (cells expressing receptor) and sources (cells expressing ligand) of the signaling communication. d) Interaction plots showing the outgoing (*APOE* expression) and incoming (ApoE receptor (Ldlr, Lrp1, etc.) expression) signal strength for each astrocyte and microglia subcluster in two sub-datasets (E3 and E4). Circle size represents the number of signaling pathways of *APOE* and its receptors among a specific cell subtype and the other cell subtypes. e) Subcluster gene expression of the four ApoE receptors expressed at detectable levels by astrocytes or microglia. f) Circos plots showing significant ApoE-receptor interactions within astrocyte and microglia subclusters for *Ldlr* (left), *Lrp8* (middle), and *Sorl1* (right). The outer circle color denotes the sending subcluster (*APOE* expressing), while the inner circle color denotes the receiving cluster(s) (a specific ApoE receptor expressing).

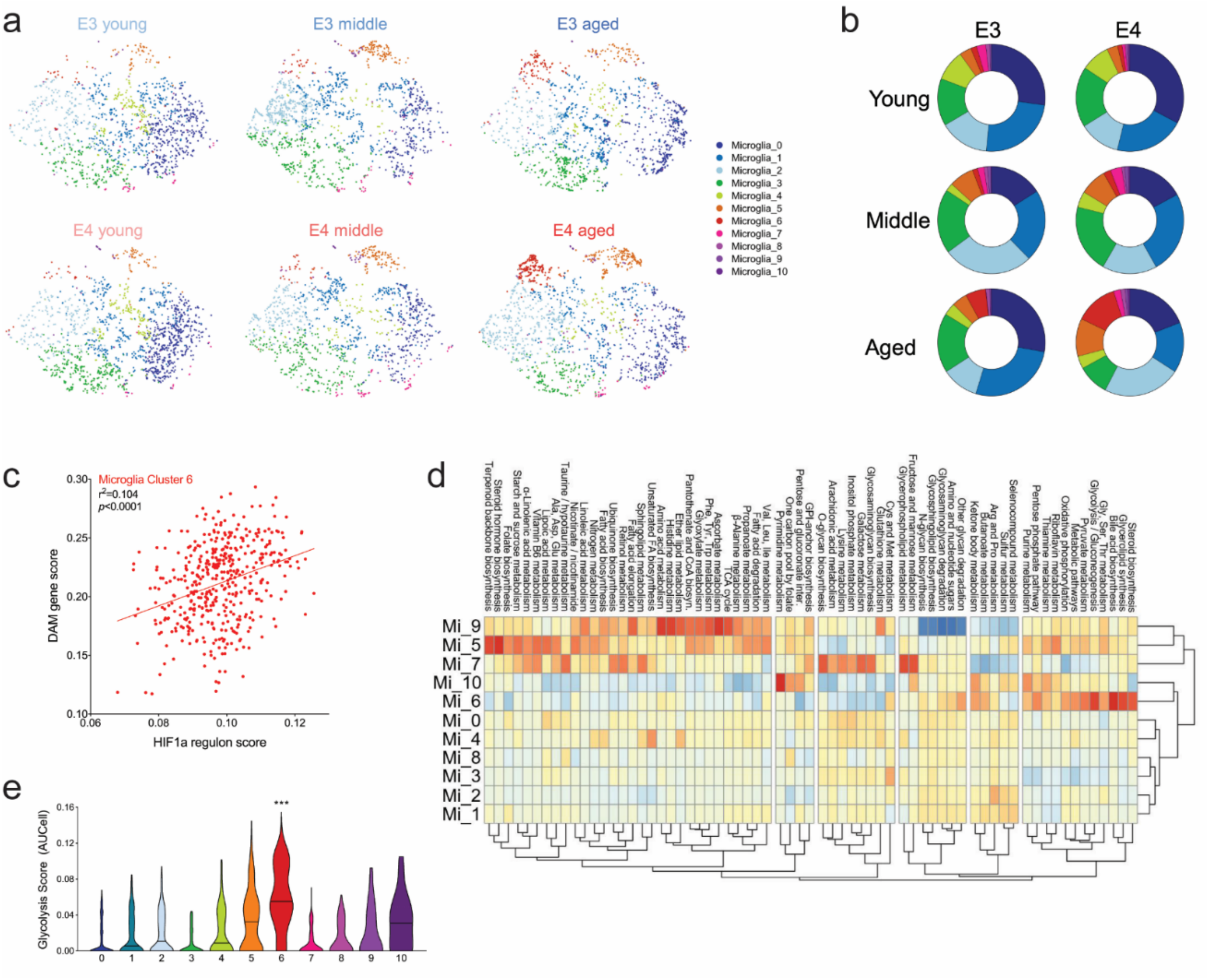

Fig. S4 (related to Fig. 2). Cell distribution and metabolic gene expression in microglia subpopulations. a-b) Distribution of microglia across clusters. a) tSNE of microglia clusters separated by experimental group. b) Donut charts showing the distribution of E3 (left) and E4 (right) microglia within each cluster across ages. c) HIF1a regulon score correlates with DAM/MgND gene scores for microglia in cluster 6. d) Heatmap showing expression of KEGG metabolic pathways in each microglia cluster. e) Glycolysis pathway scores for each microglia cluster. ***, p<0.001 Mi_6 vs all other clusters.

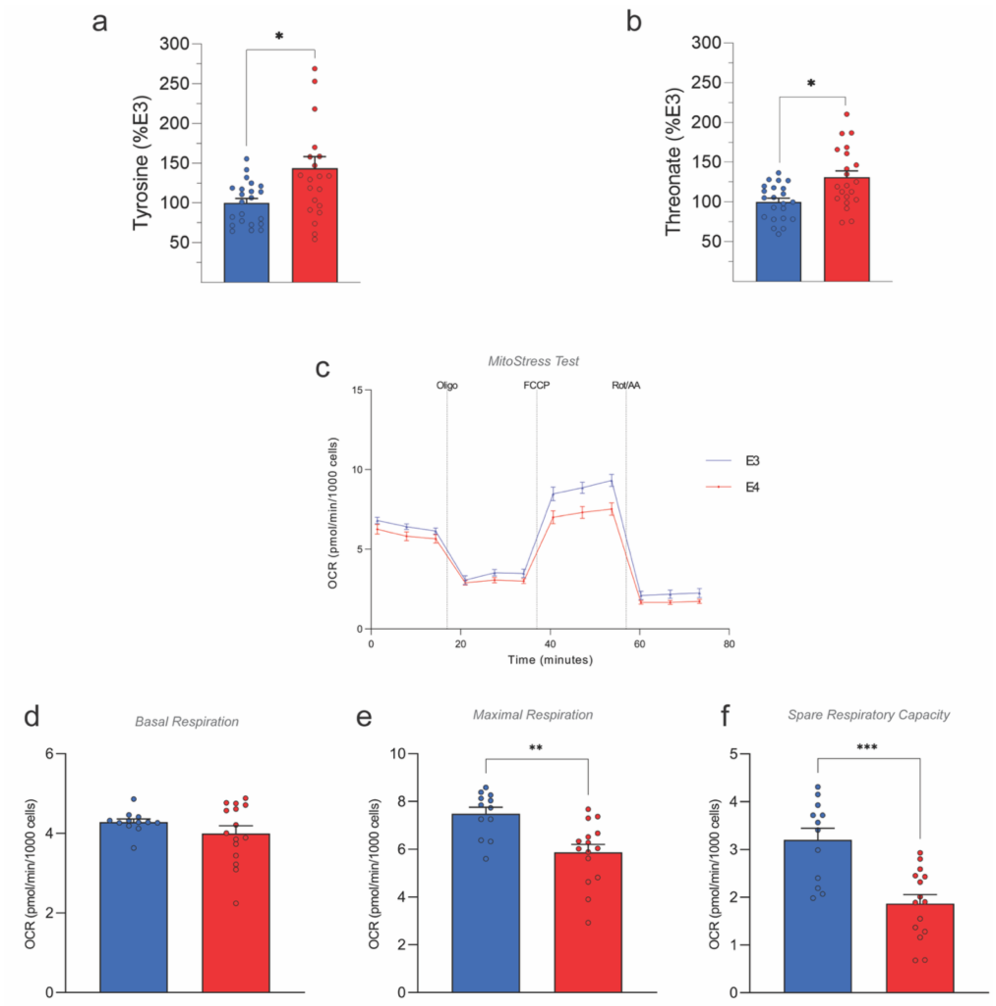

Fig. S5 (related to Fig. 4). Targeted metabolomics and oxygen consumption rate in E3 and E4 microglia. a-b) Tyrosine and threonate concentrations from targeted metabolomics of E3 and E4 microglia (*n* =21-22 per group. c-f) E3 and E4 microglia were assayed using the Seahorse Mito Stress Test (*n* =12-15 per group). c) Oxygen consumption rate (OCR) measured over time in E3 and E4 microglia. E3 and E4 microglia showed similar basal respiration (d) whereas E4 microglia showed the lowest maximal respiration (e) and spare respiratory capacity (f). $^{*}p<0.05$, $^{**}p<0.01$, $^{***}p<0.001$, two-tailed T-test.

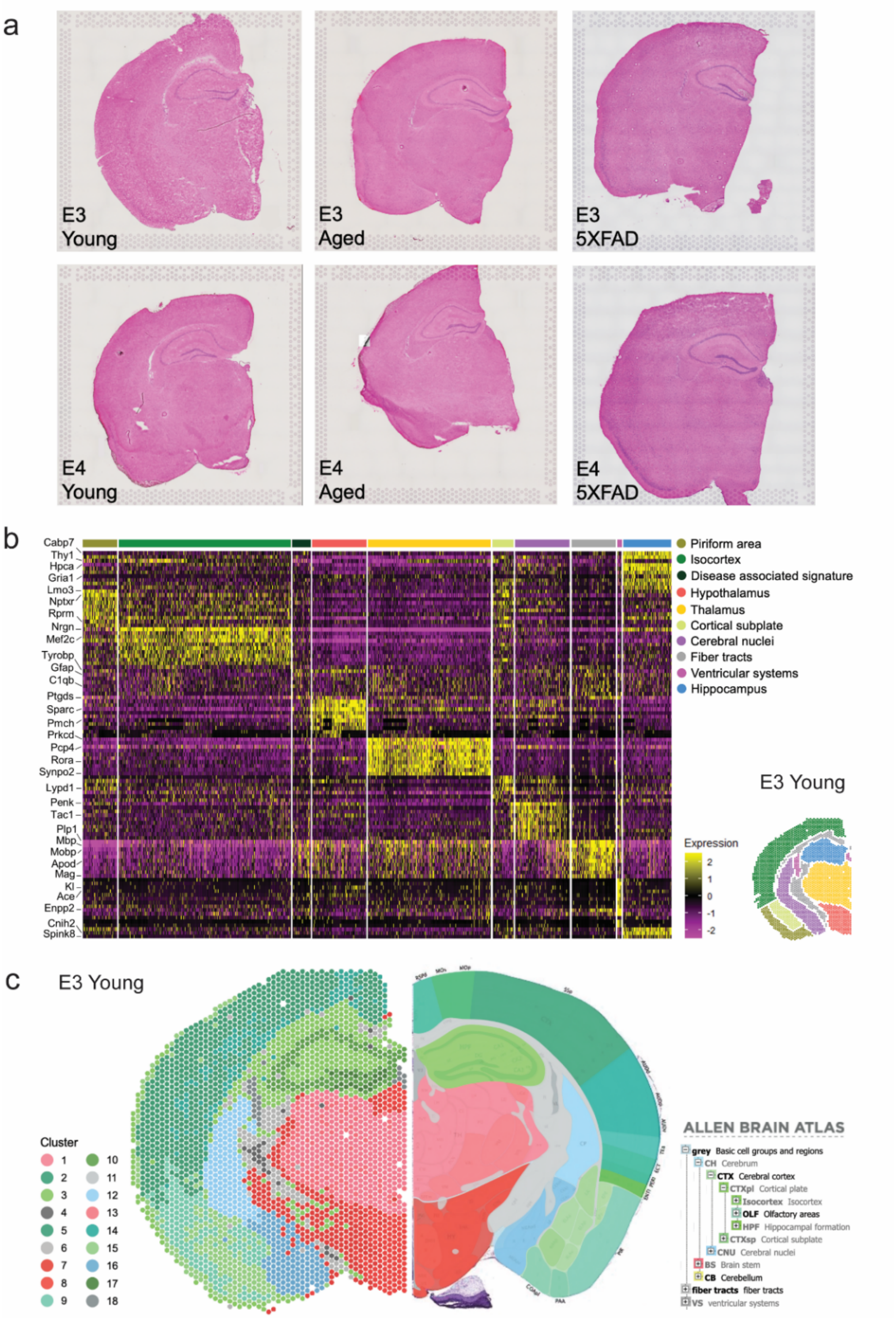

Fig. S6 (related to Fig. 5). Spatial transcriptomics shows clear clustering based on brain anatomy, plus a distinct "Disease associated signature". a) Hematoxylin and eosin (H&E) stain of coronal brain sections subjected to spatial transcriptomics (ST) analysis (Visium Spatial Gene Expression, 10X Genomics). *n* = 1 per group. b) Heat map showing expression of genes (rows) used to define the clustering of each ST spot (columns) within 9 anatomically-defined brain regions, plus the distinct "Disease associated signature" cluster. Various representative 'biomarker' genes for each cluster are shown on the left. Colored bars denote the anatomical region assigned to each cluster. Inset shows a representative brain (E3 young) color coded to reflect the assigned anatomical regions (white spots denote unassigned 'border regions'). c) Recoloring of the same 18 unbiased clusters from Figure 6 (left) to show close correspondence with anatomical brain regions of the Allen Brain Atlas (right). Representative brain shown is the 'E3 young'.

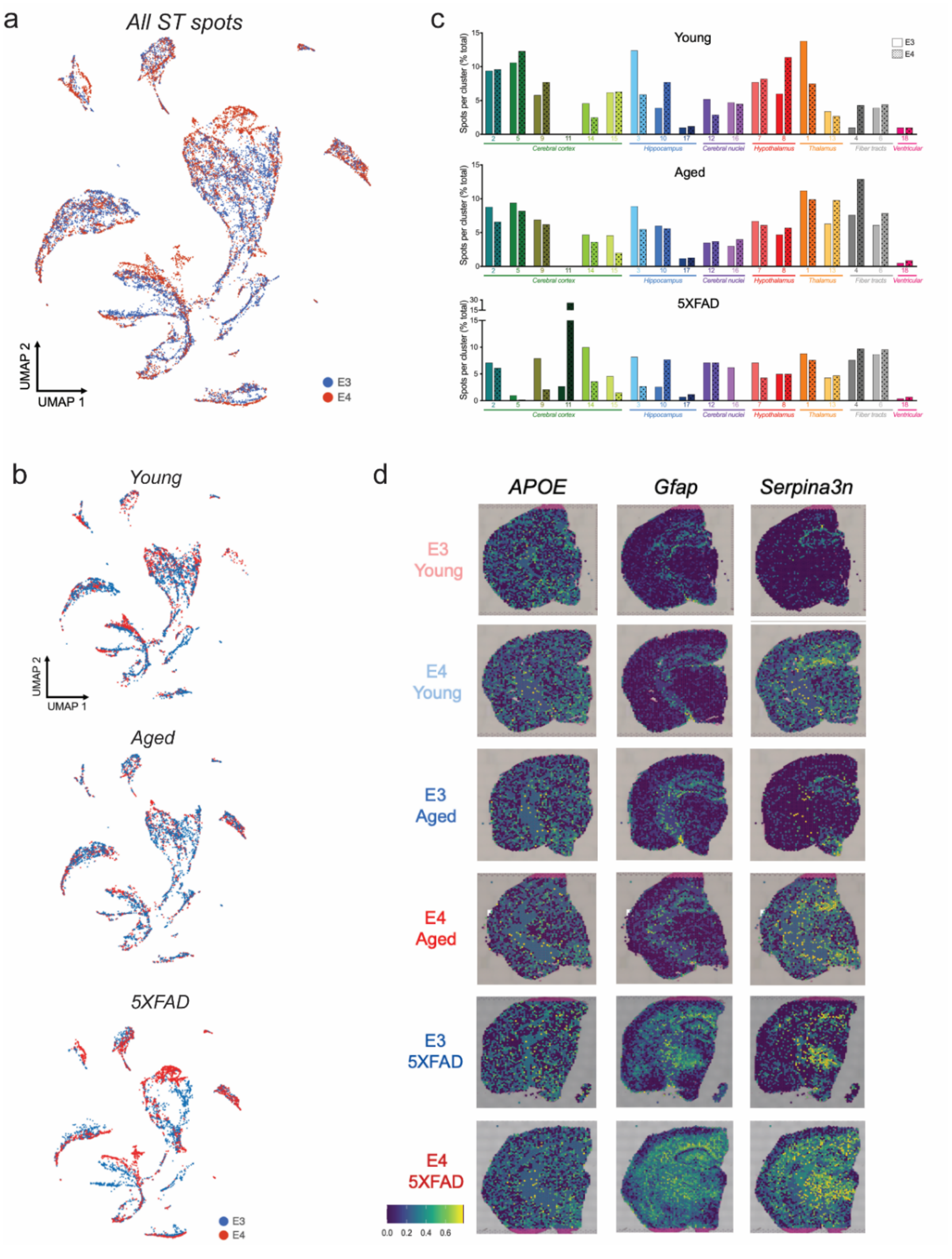

Fig. S7 (related to Fig. 5). Spatial transcriptomics UMAPs and spot number per cluster. a) UMAP showing all 79,980 spatial transcriptomic (ST) spots, colored by *APOE* genotype (E3 blue, E4 red). b) UMAPs showing ST spots from young, aged, or 5XFAD mice with E3 (blue) or E4 (red). c) Number of spots within each cluster in young, aged, or 5XFAD mice (E3 open bars, E4 dotted bars). d) Spatial gene expression of *APOE*, *Gfap* and *Serpina3n* across the brain.

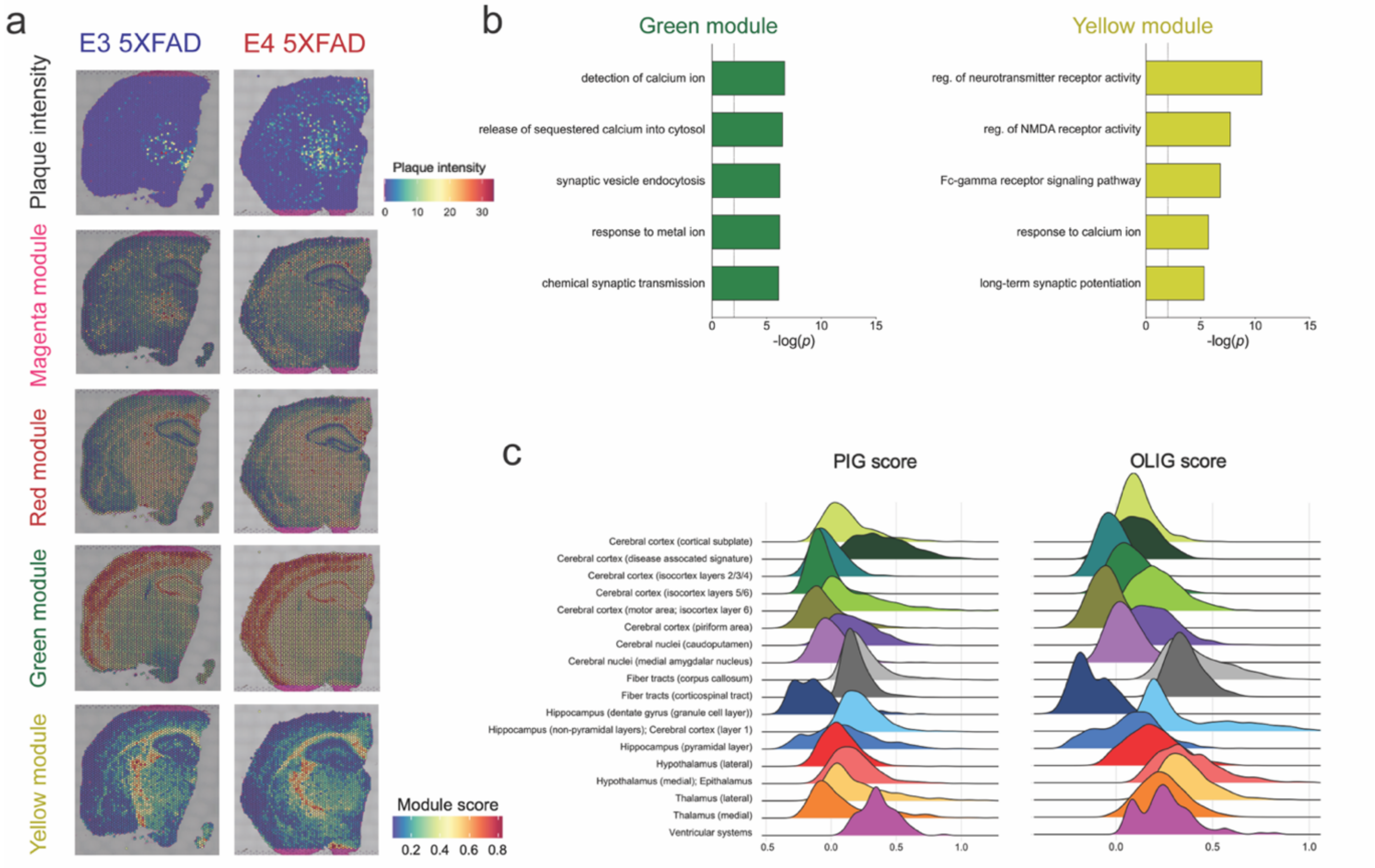

Fig. S8 (related to Fig. 6). Plaque intensity module scores, gene ontology and PIG/OLIG module scores by brain region. a) Spatial distribution of plaque intensity (top row) and spatial expression of the four gene networks (modules) significantly associated with plaque intensity in E3 5XFAD (left) and E4 5XFAD (right) brains. b) Top 5 gene ontology terms associated with the two modules (green, yellow) negatively correlated with plaque intensity. c) Ridge plots showing PIG and OLIG module scores for each spatial transcriptomics cluster (with corresponding anatomical assignment). Module scores are composite scores for all 6 spatial transcriptomics brains combined.

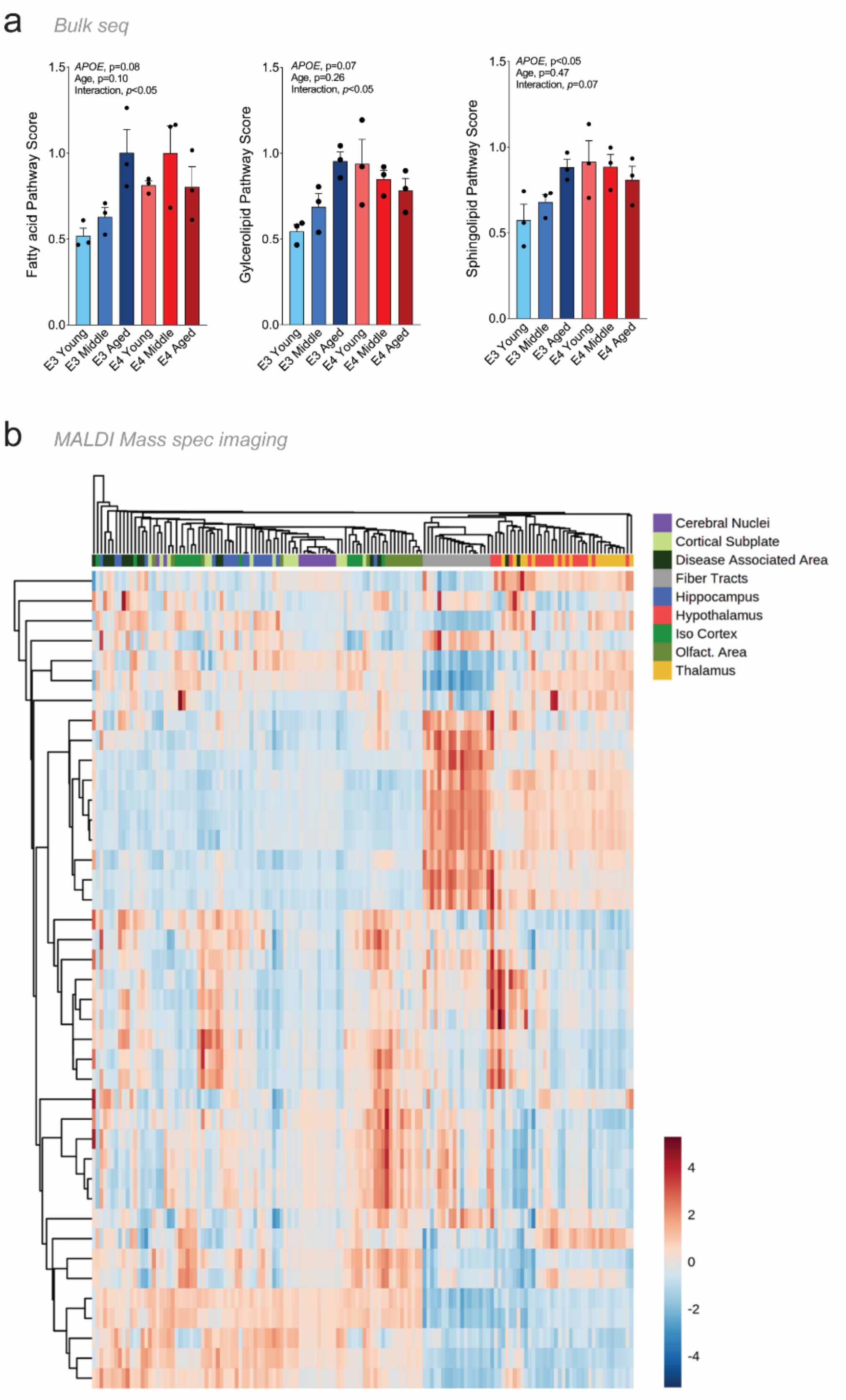

Fig. S9 (related to Fig. 7). Lipid metabolism pathways from bulk sequencing, MALDI MSI quantification of lipids by brain region. a) Expression of fatty acid, glycerolipid, and sphingolipid pathway genes increase with *APOE* and/or age in whole brain tissue. b) Targeted lipidomics via MALDI MSI shows clear clustering by brain region, with the exception of the cortical and subcortical "disease associated area" found primarily in E4 5XFAD mice. Heat map displaying intensity of lipids (rows) quantified within each brain region (column). *n* =3 mice per group.
